# miR-206 integrates multiple components of differentiation pathways to control the transition from growth to differentiation in rhabdomyosarcoma cells

**DOI:** 10.1186/2044-5040-2-7

**Published:** 2012-04-29

**Authors:** Kyle L MacQuarrie, Zizhen Yao, Janet M Young, Yi Cao, Stephen J Tapscott

**Affiliations:** 1 Human Biology Division, Fred Hutchinson Cancer Research Center, 1100 Fairview Ave N, C3-168, Seattle, WA, 98109, USA; 2 Molecular and Cellular Biology Program, University of Washington, Seattle, WA, 98105, USA; 3 Division of Basic Sciences, Fred Hutchinson Cancer Research Center, Seattle, WA, 98109, USA; 4 Department of Neurology, University of Washington, Seattle, WA, 98105, USA

**Keywords:** Rhabdomyosarcoma, RUNX1, ZNF238, miR-206, MSC, myogenesis

## Abstract

**Background:**

Similar to replicating myoblasts, many rhabdomyosarcoma cells express the myogenic determination gene MyoD. In contrast to myoblasts, rhabdomyosarcoma cells do not make the transition from a regulative growth phase to terminal differentiation. Previously we demonstrated that the forced expression of MyoD with its E-protein dimerization partner was sufficient to induce differentiation and suppress multiple growth-promoting genes, suggesting that the dimer was targeting a switch that regulated the transition from growth to differentiation. Our data also suggested that a balance between various inhibitory transcription factors and MyoD activity kept rhabdomyosarcomas trapped in a proliferative state.

**Methods:**

Potential myogenic co-factors were tested for their ability to drive differentiation in rhabdomyosarcoma cell culture models, and their relation to MyoD activity determined through molecular biological experiments.

**Results:**

Modulation of the transcription factors RUNX1 and ZNF238 can induce differentiation in rhabdomyosarcoma cells and their activity is integrated, at least in part, through the activation of miR-206, which acts as a genetic switch to transition the cell from a proliferative growth phase to differentiation. The inhibitory transcription factor MSC also plays a role in controlling miR-206, appearing to function by occluding a binding site for MyoD in the miR-206 promoter.

**Conclusions:**

These findings support a network model composed of coupled regulatory circuits with miR-206 functioning as a switch regulating the transition from one stable state (growth) to another (differentiation).

## Background

Rhabdomyosarcoma (RMS) is a soft tissue sarcoma characterized by expression of myogenic regulatory factors, especially *MyoD*, and other skeletal muscle genes [1-3]. *MyoD* is capable of converting multiple cell types into terminally differentiated skeletal muscle [4,5] and normally acts as a nodal point in differentiation to integrate multiple signals to balance regulative growth and cell differentiation [6]. However, in RMS the ability of MyoD to induce differentiation is impaired [7].

Our recent study indicated that rhabdomyosarcomas represent an arrested progress through a normal transitional state that is regulated by the formation of heterodimers between MyoD and the full-length E-proteins [8]. MyoD binds DNA as a heterodimer with an E-protein (E2A, E2-2, or HEB) [9]. Normal myoblasts and RMS express a transcriptionally less active splice form of E2A as well as the bHLH (basic helix-loop-helix) proteins ID and Musculin (MSC), both of which compete with the full-length E-proteins for heterodimerization with MyoD. The demonstration that a forced heterodimer of MyoD and a full-length E-protein suppressed multiple inhibitory mechanisms and induced differentiation in the RD and other rhabdomyosarcomas suggested that a central integrating mechanism might regulate the switch from regulative growth to differentiation [8].

If a central integrating mechanism does exist, then multiple pathways should regulate its activity and multiple factors should be able to induce differentiation in RMS. In this manuscript we demonstrate that modulation of multiple different myogenic factors can induce differentiation in rhabdomyosarcoma cells and that their activity is integrated, at least in part, through the activation of miR-206, which acts as a genetic switch to transition the cell from a proliferative growth phase to differentiation. These findings suggest that multiple components of differentiation pathways that converge on miR-206 might be targeted for differentiation therapies in at least some rhabdomyosarcomas.

## Methods

### Plasmid construction

Coding sequences of RUNX1 and ZNF238 were amplified by PCR from human myotube cDNA, and cloned into pRRLSIN.cPPT.PGK/GFP.WPRE, pCLBabe, and pCS2. The miR-206 lentivirus was purchased from Open Biosystems. Lentiviral supernatant was produced by the FHCRC core viral facility, and viruses from pCLBabe plasmids packaged using BBS-mediated calcium precipitation into Phoenix cells. MD ~ E2/5 was cloned into the pCLBabe backbone. For the miR-206 promoter luciferase reporter, a piece of approximately 2.5 kb of DNA upstream of human miR-206 was amplified using primers located in ( Additional file 1: Table S1).

### Cell culture, transient transfections, and luciferase assays

RD cells were obtained from ATCC (American Type Culture Collection) in approximately 1990, and all analyses have been performed on cells that originated from low passage number frozen aliquots. RhJT cells were obtained from PJ Houghton in 1990 and, as with RD cells, all experiments have used cells from original low passage number frozen cells. RD and RhJT cells were maintained in DMEM with 10% bovine calf serum and 1% Pen-Strep (Gibco, Grand Island, USA). Low-serum differentiation media consisted of DMEM with 1% horse serum, 1% Pen-Strep and 10 μg/mL insulin and transferrin. Transient transfections for luciferase assays were performed using Superfect according to manufacturer’s directions with a total of 3 ug of plasmid DNA in each well (Qiagen, Valencia, USA). Luciferase assays used the Dual-Luciferase Assay kit (Promega, Madison, USA) according to manufacturer’s directions. All results were corrected to co-transfected Renilla-pCS2 and are reported as the mean ± SEM of at least three independent experiments, with significance calculated using a *t*-test, and each experiment having three biological replicates of all conditions. Transient transfections of pre-microRNA constructs were performed using 25 μM of pre-miR constructs (Ambion, Grand Island, USA) and siPORT NeoFX (Ambion) according to the manufacturer’s directions.

### qPCR and RT-PCR

All qPCR was performed using SybrGreen from Bio-Rad on an Applied Biosystems 7900HT. Relative expression levels were calculated using cDNA dilution standard curves or delta-delta Ct calculations. All values are reported as the mean ± SEM of at least three independent biological experiments and significance tested with t-tests. All RT-PCRs were performed simultaneously with minus reverse transcriptase controls to check the absence of signal. Primers used for amplification are listed in ( Additional file 1: Table S1).

### microRNA microarrays

RNA was isolated using acid-phenol purification from two biologically independent sets of RD cells transduced with either MD ~ E or empty vector pCLBabe retroviruses and differentiated for 24 h after puromycin selection. miRNAs were labeled using Exiqon’s miRCURY labeling kit, and then competitively hybridized to in-house spotted miRNA arrays (FHCRC core facility). Cutoffs for significant changes were a FDR <0.05 and a fold-change >2. Data are available under GEO accession number GSE35921.

### microRNA northerns

microRNA northern blots were performed as described previously [10]. Probe sequences used are listed in ( Additional file 1: Table S1).

### Expression microarrays

RNA was isolated using the RNeasy mini kit (Qiagen) from RD cells infected with either RUNX1-, ZNF238-, miR-206-, or GFP-expressing lentiviruses and allowed to differentiate for 72 h. Each condition was performed with three independent biological replicates. RNA was hybridized to Illumina Human HT-12 v4 BeadChips. Analysis was performed in R/Bioconductor using the lumi and limma packages with annotations found in the lumiHumanAll.db package. *P* values were adjusted to account for multiple testing using Benjamini and Hochberg’s method, and cutoffs for significant changes were a FDR <0.05 and a fold-change >2. GO category enrichment tests were performed using the conditional algorithm of the GOstats package and a gene ‘universe’ of any gene with a GO annotation that was called as ‘present’ in at least one of the 12 array datasets. Data are available under GEO accession number GSE35921.

### ChIPs (Chromatin immunoprecipitations)

ChIPs were performed as has been described previously [11]. Antibodies used were as follows: RUNX1 (Abcam, ab23980), MyoD [12], MSC (Santa Cruz, sc-9556X), Acetylated Histone H4 (Upstate 06-866). Primers used for amplification are listed in ( Additional file 1: Table S1).

### EdU and BrdU labeling, western blots, and cell stains

After 24 h in low-serum differentiation media, cells were shifted to differentiation media supplemented with EdU at a final concentration of 50 μM (Invitrogen) and incubated for a further 24 h. Cells were then fixed and stained according to the manufacturer’s protocols using the Click-iT kit, and total nuclei and EdU positive nuclei counted by hand.

RD cells were labeled with BrdU at a final concentration of 30 μg/mL in differentiation media for 24 h. Cells were treated with hydrochloric acid before incubation with anti-BrdU antibody (Invitrogen A21300), and fluorescent secondary antibody (Jackson Immunoresearch). Nuclei were detected with DAPI, and cells counted by hand.

Western blots were performed on whole cell lysates collected in Laemelli buffer containing 10% beta-mercaptoethanol. All blots were blocked in 3% milk (w/v) in 0.5% Tween-20-containing PBS before incubation with primary antibody (MHC: MF-20, MyoD: 5.8A, RUNX1: Abcam, ab23980), a HRP-conjugated secondary antibody (Jackson, West Grove, USA), and chemiluminescent detection (Amersham, Pittsburgh, USA).

Cells were fixed with 2% paraformaldehyde for 6 min at room temperature before permeabilization with Triton X-100. Myosin heavy chain was detected with the MF-20 antibody, and nuclei detected with DAPI.

### Electrophoretic mobility shift assays

Electrophoretic mobility shift assays were performed as described previously [13]. Probe sequences are listed in ( Additional file 1: Table S1).

## Results

### RUNX1 and ZNF238 differentiate rhabdomyosarcoma cells

**Figure 1:**
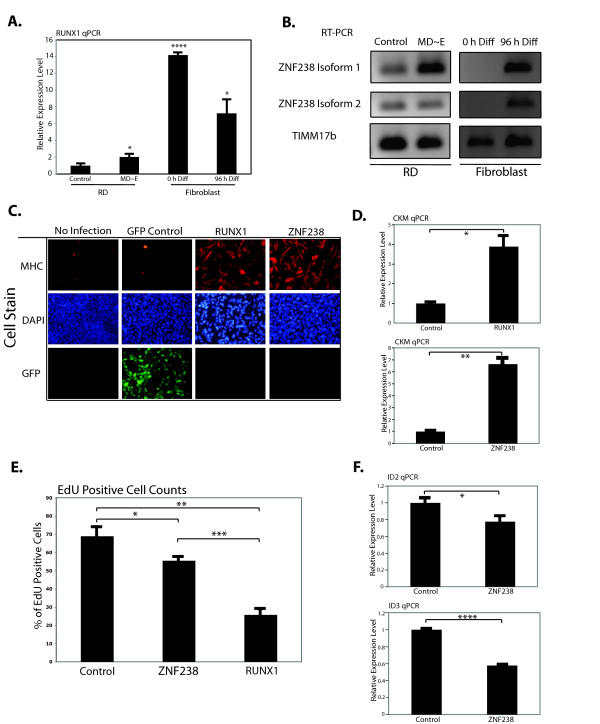
**Expression of* RUNX1 *or* ZNF238 *leads to terminal differentiation of RMS cells.** (**A**) qPCR for *RUNX1* was performed in RD cells either infected with a control virus, or the forced MyoD ~ E dimer (MD ~ E) as well as control (0 h) human fibroblasts and fibroblasts differentiated into myotubes (96 h). (**B**) RT-PCR for the two isoforms of *ZNF238* in RD cells and fibroblasts as in 1A. (**C**) Myosin heavy chain (MHC) immunostains in RD cells either not infected (no infection), infected with a control GFP-expressing lentivirus (GFP control) or *RUNX1* or *ZNF238* expressing lentivirus. All cells were infected at approximately equivalent MOIs, and cells were allowed to differentiate for 72 h in low-serum media before staining. GFP was detected directly, without the use of an antibody. (**D**) qPCR for muscle-specific creatine kinase (*CKM*) in RD cells infected with either ZNF238 or RUNX1 viruses compared to cells with control retroviruses. (**E**) After 24 h of differentiation, RD cells were pulsed for a further 24 h with EdU-containing differentiation media, before fixation and quantification of the percentage of EdU-positive cells. (**F**) qPCR for *ID2* and *ID3* in control and *ZNF238*-expressing RD cells. All qPCR data are normalized to *TIMM17b* expression, and the level in control cells is set to 1. All bar graphs represent the mean ± SEM of at least three independent experiments. *: *P* < 0.05; **: *P* < 0.01; ***: *P* < 0.001; ****: *P* < 1 × 10^-4^.

*RUNX1* is a runt-related transcription factor with a well-characterized hematopoietic role [14] that is expressed in muscle cells [15], functions in denervated muscle [16], and is regulated by the MyoD network [8,17], but has an uncharacterized role in developing muscle. *ZNF238* (aka *RP58*) is induced by MyoD and directly down-regulates the inhibitory *Id* factors [18]. MyoD ChIP-seq (chromatin immunoprecipitation coupled to high-throughput sequencing) found an association between the RUNX1 and ZNF238 binding motifs and MyoD bound sites in muscle cells [11] and qPCR confirmed that both *RUNX1* and *ZNF238* increased with differentiation induced in the RD RMS line by the forced MD ~ E dimer and that myotubes formed from MyoD-induced myogenesis in normal fibroblasts had higher expression levels of both factors compared to RD cells (Figure 1A and 1B). Therefore, we hypothesized that RUNX1 and/or ZNF238 might cooperate with MyoD to drive muscle gene expression and tested each factor for its ability to induce differentiation in RD cells.

Lentiviral-mediated expression of each factor in RD cells ( Additional file 2: Figure S1) induced muscle differentiation, as measured by morphology and expression of myosin heavy chain (MHC) (Figure 1C), muscle-specific creatine kinase (*CKM*) (Figure 1D), and withdrawal from the cell cycle based on EdU incorporation (Figure 1E). As in normal myogenesis, expression of *ZNF238* decreased both *ID2* and *ID3* (Figure 1F). This differentiation is not restricted to the embryonal RMS RD cell line; the RhJT alveolar line expressing RUNX1 showed similar results ( Additional file 3: Figure S2).

### miR-206 expression correlates with factor-induced differentiation in RMS cells

**Figure 2:**
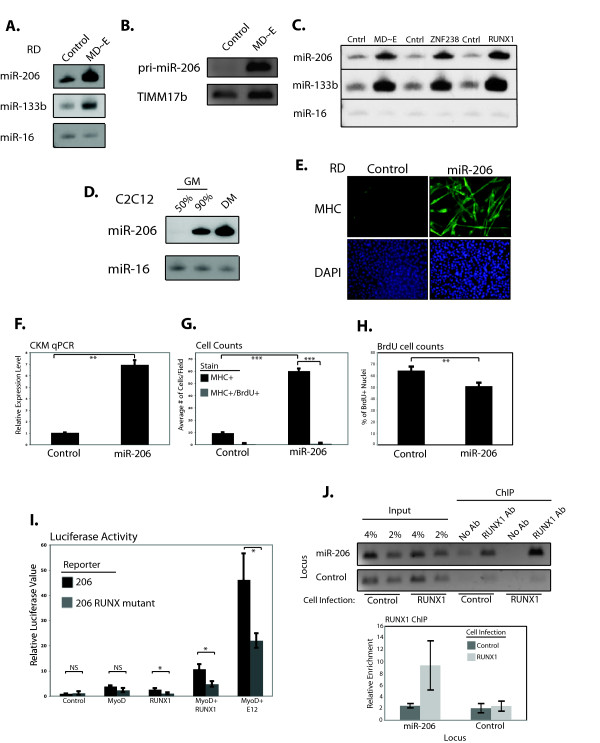
**MyoD ~ E, RUNX1, and ZNF238 increase miR-206.** (**A**) microRNA northern blots to detect the mature form of the indicated microRNAs in RD cells infected with either empty (control) retrovirus, or retrovirus expressing MyoD ~ E (MD ~ E). (**B**) RT-PCR using primers located in the pre- and pri-miR-206 sequence to detect the primary miR-206 transcript. *TIMM17b* is the internal control. (**C**) microRNA northerns as in 2A, in RD cells expressing a transcription factor as indicated. (**D**) microRNA northerns in C2C12 cells at various stages of differentiation ranging from undifferentiated myoblasts (50% GM), through beginning differentiation (90% GM) to myotubes (DM). (**E**) Immunostains for MHC in RD cells transiently transfected with either a pre-miR-206 RNA construct, or a negative control construct. Nuclei were stained with DAPI. (**F**) qPCR for *CKM* in RD cells treated as in E. (**G**) RD cells treated as in E were pulsed with BrdU for 24 h and then stained and counted to determine the extent of co-localization of MHC + myotubes and BrdU + nuclei. (**H**) RD cells transiently transfected as in E were pulsed for 24 h with BrdU-containing differentiation media before fixation and quantification of the percentage of BrdU positive cells. The percent reduction of BrdU + nuclei almost exactly equals the percent of cells found to be MHC + (not shown). (**I**) Luciferase activity in RD cells using a miR-206 promoter driven reporter and transiently transfected factors as indicated. ‘206 RUNX mutant’ indicates that the reporter has had a putative RUNX1 binding site mutated to prevent RUNX1 binding. Control indicates transfection with an empty plasmid. All luciferase assays were normalized to the results from a co-transfected renilla plasmid. (**J**) RUNX1 ChIP assays, both with normal PCR and qPCR results, at the miR-206 promoter and a control locus before (Control) and after (RUNX1) infection of the cells with empty or *RUNX1*-expressing retrovirus. All PCRs in the imaged gel (upper) were performed for the same number of cycles. qPCR results (lower) represent the mean ± SEM of two independent ChIP experiments. Relative enrichment is calculated as the ratio of the % of input amplified with antibody to the % of input amplified with no antibody. All other graphs in this figure represent the mean ± SEM of at least three independent experiments. * : *P* < 0.05; ** : *P* < 0.01; *** : *P* < 0.001.

We previously demonstrated that the expression of the MD ~ E dimer in RD cells down-regulated multiple myogenic inhibitors [8], consistent with induction of a microRNA. MicroRNA microarrays from MD ~ E transduced RD cells identified several microRNAs that changed expression and miR-206, a microRNA that has been shown to induce myogenic differentiation in normal cells and RMS [19,20], was the most consistently increased ( Additional file 4: Table S2) and was confirmed by miRNA Northern blotting (Figure 2A, upper panel), as was miR-133b, a miRNA from the same primary transcript (Figure 2A, second panel). RT-PCR for the presumptive primary transcript also showed a substantial increase (Figure 2B). Northern blotting confirmed that the forced dimer decreased miR-199a* expression, as suggested by the array results ( Additional file 5: Figure S3A), and that miR-29b, previously implicated in RMS differentiation, was expressed at low levels and did not change in response to MD ~ E expression ( Additional file 5: Figure S3B) [21]. Furthermore, RD cells differentiated through *RUNX1* and *ZNF238* expression showed an increase in mature miR-206 in both cases (Figure 2C), along with an increase in primary transcript (data not shown). miR-206 levels in the myogenic C2C12 cell line showed that miR-206 expression in proliferative and differentiated RMS resembled the changes as C2C12 cells shift from beginning myogenesis (90% confluency) to myotubes (DM) (Figure 2D).

In agreement with previous reports demonstrating that miR-206 alone is sufficient to differentiate RMS cells [20,22,23], transient transfection of pre-miR-206 constructs into RD cells resulted in myotube formation (Figure 2E), an increase in *CKM* expression (Figure 2F), and evidence that cells undergoing morphological change withdrew from the cell cycle (Figure 2G, H), with similar results in the alveolar RhJT cells ( Additional file 6: Figure S4A and data not shown). As would be expected from prior reports of its effect on myogenic cells [24], introduction of miR-133b did not lead to RMS differentiation as judged by either morphology or gene expression ( Additional file 6: Figure S4B, C).

MyoD directly regulates miR-206 [25] and a putative RUNX1 binding site exists near the MyoD binding site. RUNX1 alone showed minor activation of a miR-206 luciferase reporter, while RUNX1 combined with MyoD showed synergistic activation (Figure 2I, black bars), which was dependent on the integrity of the RUNX1 binding site (Figure 2I, grey bars). ChIP experiments in RD cells confirmed that RUNX1 binds the miR-206 promoter (Figure 2J). Therefore, RUNX1 cooperates with MyoD to enhance the expression of miR-206. ZNF238 did not activate the miR-206 reporter (data not shown), suggesting that the reporter does not have the elements or context for ZNF238 regulation.

**Figure 3:**
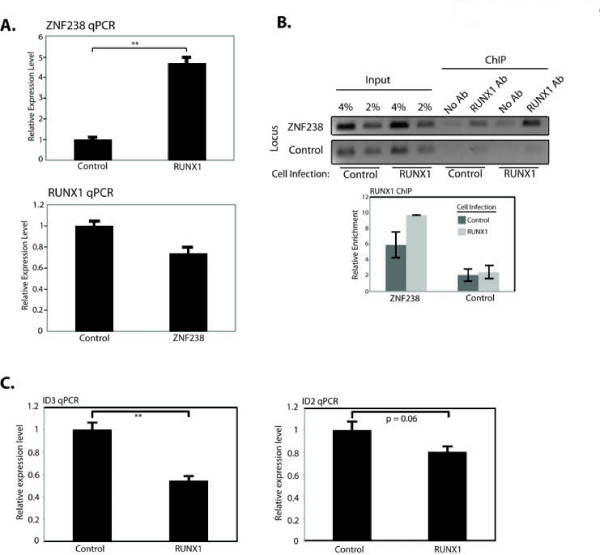
**ZNF238 is a downstream target of MyoD and RUNX1.** (**A**) qPCR for *RUNX1* and *ZNF238* in RD cells transduced with virus expressing the converse factor. (**B**) RUNX1 ChIP results at the intron of *ZNF238* and a control locus before (Control) and after (RUNX1) infection of the cells with empty or *RUNX1*-expressing retrovirus. (**C**) qPCR for *ID* genes in control and RUNX1-expressing RD cells. PCRs in the imaged gel in 3B were performed for the same number of cycles. The graph in 3B represents qPCR data showing the mean ± SEM of two independent experiments. Relative enrichment is calculated as the ratio of the % of input amplified with antibody to the % of input amplified with no antibody. qPCRs in 3A and 3C are represented as the mean ± SEM of at least three independent experiments. *: *P* < 0.05; **: *P* < 0.01.

RUNX1 also induced the expression of *ZNF238* in RD cells and ChIP identified RUNX1 binding close to the TSS of *ZNF238*, suggesting it functions directly to activate *ZNF238* (Figure 3A and 3B). Consistent with this model, ID2 and ID3 expression were decreased in RD cells expressing RUNX1 (Figure 3C). In contrast, ZNF238 did not upregulate *RUNX1* expression, but we confirmed the prior report [18] that MyoD activates *ZNF238* ( Additional file 7: Figure S5). Together, this shows that MyoD functions in nested feed-forward circuits with RUNX1 and ZNF238 to activate miR-206 expression and induce differentiation in the RD cells.

### RUNX1, ZNF238, and miR-206 activate a common differentiation program

**Figure 4:**
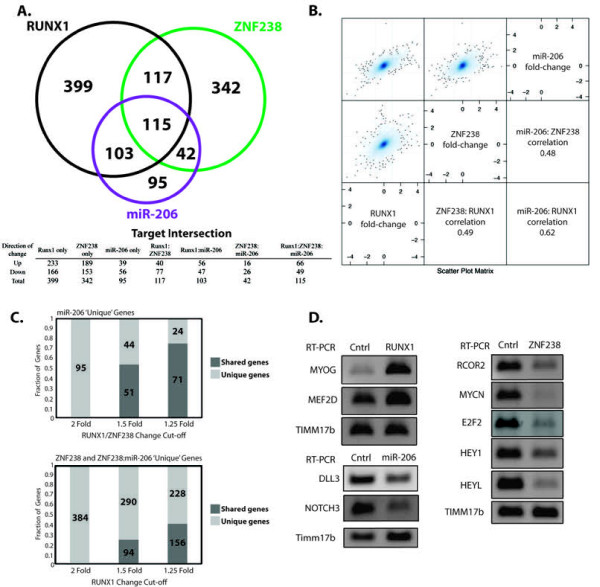
**RUNX1, ZNF238, and miR-206 function through common mechanisms.** (**A**) 3-way Venn diagram representing the overlap between significantly regulated (fold-change >2, FDR <0.05, overlap considers only changes in same direction) gene targets in RD cells differentiated either through *RUNX1*, *ZNF238*, or miR-206 expression relative to GFP-infected controls. The table indicates the breakdown of upregulated versus down-regulated genes for each portion of the Venn diagram. (**B**) Scatter plots showing pairwise comparisons of gene expression. All values are plotted as the log_2_ of the fold-change relative to GFP-infected controls, as indicated along the x- and y-axes. Correlation is listed for each comparison in the matrix. (**C**) (upper panel) Bar graph demonstrating that the majority of the 95 genes listed as being ‘uniquely’ regulated by miR-206 in 4A are also regulated by RUNX1 and/or ZNF238, but at lower levels of expression change. FDR was kept constant (<0.05) in this analysis, and to be included as a ‘shared’ target, the change had to occur in the same direction (either up- or down-regulated) in RUNX1 and/or ZNF238 as in miR-206. (bottom panel) Analysis as in the top panel for genes in the ZNF238 unique and ZNF238:miR-206 intersection groups relative to RUNX1 changes. (**D**) RT-PCR for select gene targets from Additional file 9: Table S4. *TIMM17b* serves as the internal control.

Gene expression arrays were performed on RD cells transduced individually with *RUNX1-*, *ZNF238-*, and miR-206 expressing viruses. GO analysis ranked by *P* values identified multiple muscle-related categories induced by each factor, with four of the ten most significant categories shared between all factors ( Additional file 8: Table S3). In agreement with our deduced epistatic relationship, the number of genes significantly regulated (fold change > 2, FDR <0.05) by each factor became sequentially reduced from RUNX1 (734) to ZNF238 (616) to miR-206 (355) and showed substantial overlap (Figure 4A) and correlation (Figure 4B). Loosening the fold-change threshold showed that nearly all genes regulated by miR-206 similarly changed expression in response to RUNX1 and/or ZNF238 (Figure 4C, top). Similarly, a large portion of the ZNF238 genes were also regulated by RUNX1 (Figure 4C, bottom).

RUNX1, ZNF238, and miR-206 affected the expression of a variety of transcription factors and signaling pathways involved in myogenesis ( Additional file 9: Table S4), and a subset of the changes were confirmed by RT-PCR (Figure 4D). The MRF myogenin, a MyoD target [26], increased in response to all three of the factors. *MEF2C* and *MEF2D*, cooperative factors for MyoD activity [17], were up-regulated. Down-regulation of positive regulators of the cell cycle (*MYCNRCOR2E2F2*) and various members of the *HES/HEY* family (*HEY1, HES6, HEYLHES1*) was observed as well. It has previously been demonstrated that interference with HES1 contributes to RMS proliferation [27], and the *HES/HEY* family is known to be Notch responsive [28], a signaling pathway with myogenic inhibitory effects [29-31]. Among miR-206’s most strongly down-regulated targets were two members of the Notch signaling pathway, *DLL3* and *NOTCH3*.

### The miR-206 promoter integrates multiple inputs to switch from inhibition by MSC to activation by MyoD

We have previously shown that MyoD binds and activates the expression of the miR-206 gene in normal myogenesis [25], whereas our current data indicate that the miR-206 promoter integrates multiple inputs to modulate its activation by MyoD. Somewhat to our surprise, ChIP in RD cells showed that MyoD was bound to the miR-206 promoter in undifferentiated RD cells that expressed only low levels of miR-206 ( Additional file 10: Figure S6A) and regional enrichment for acetylated H4 histones indicated active regional histone acetyltransferase activity ( Additional file 10: Figure S6B). Therefore, a factor, or factors, was preventing efficient transcriptional activation by the bound MyoD.

**Figure 5:**
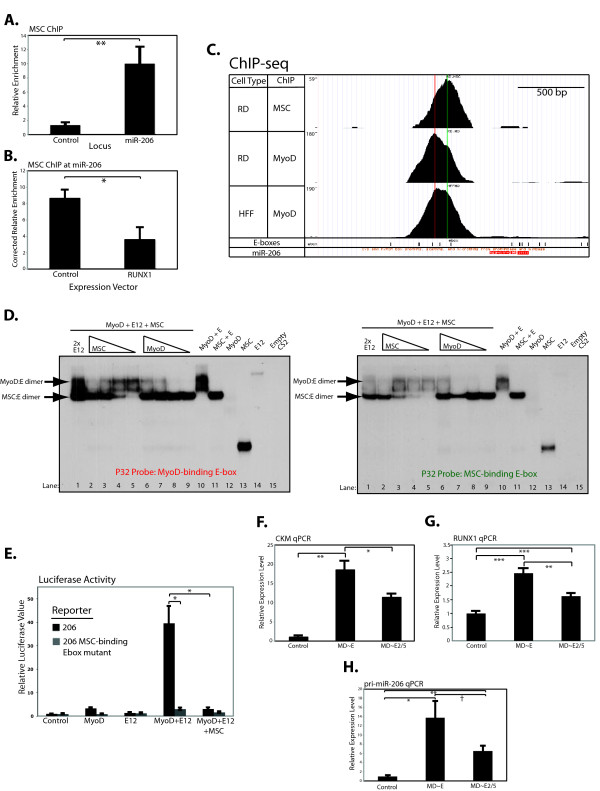
**MSC interferes with the ability of MyoD to positively regulate the microRNA miR-206 by blockading a necessary MyoD-binding site.** (**A**) Site-specific ChIP for MSC in the miR-206 promoter and at a control locus. (**B**) MSC ChIP as in (A) in RD cells infected either with an empty retrovirus (Control), or RD cells differentiated through expression of *RUNX1* (RUNX1). (**C**) Screenshot from the human UCSC Genome Browser of the region that corresponds to the miR-206 promoter. Mapped reads from ChIP-seq for MyoD in RD and HFF cells are indicated, with the number on the left-hand y-axis indicating the number of reads mapped at the peak of occupancy. The location of E-boxes are indicated at the bottom of the panel by the black rectangles. Vertical lines are drawn through the apparent highest points of occupancy for MyoD (red) and MSC (green) in RD cells. (**D**) Electrophoretic mobility shift assays using *in vitro* transcribed and translated proteins as indicated and probes that correspond to either the E-box located at the peak of MyoD binding as indicated by the red mark in 5C, or the E-box located at the peak of MSC binding, indicated by the green mark. The position of MyoD:E and MSC:E heterodimers are indicated by the arrows. The lane marked 2x E12 indicates protein mixtures that included double the amount of E12 compared to other lanes, and the triangles indicate decreasing amounts of either MSC or MyoD in the mixtures as other proteins were maintained at constant levels and total protein amounts were balanced with translation of empty CS2. (**E**) Luciferase assays in RD cells with constructs as indicated below the figure using either the miR-206 promoter luciferase reporter (206) or one in which the E-box that the peak of MSC occupancy is located over has been mutated (206 MSC-binding Ebox mutant). Control indicates transfection with an empty plasmid. All luciferase assays were normalized to the results from a co-transfected renilla plasmid. (**F**) qPCR for *CKM* from RD cells transduced with either an empty virus (control), or one expressing either the MD ~ E or MD ~ E2/5 forced dimer. Cells were differentiated for 24 h before collection of RNA for use in qPCR. (**G**) qPCR for *RUNX1* from the RD cells assayed in E. (**H**) qPCR for pri-miR-206 from the RD cells assayed in E and F. For all ChIPs, relative enrichment is calculated as the ratio of the % of input amplified with antibody to the % of input amplified with no antibody. The control locus is located at hemoglobin beta, a silent gene in myogenic cells. Corrected relative enrichment (5B) is the ratio of enrichment at miR-206 to enrichment at the control locus. All graphs represent the mean ± SEM of at least three independent experiments. All qPCR of gene expression was corrected to *TIMM17b* and control cells set to 1. * : *P* <0.05; ** : *P* < 0.01; *** : *P* < 0.001; † : *P* = 0.058.

We have previously shown that the inhibitory bHLH factor Musculin (MSC) inhibits MyoD activity in RD cells and other RMS [8]. Furthermore, since miR-206 has been shown to inhibit *MSC* expression [19], we hypothesized that the opposing activities of MyoD and MSC might constitute an on-off switch at the miR-206 promoter. ChIP showed that MSC was bound to the same region as MyoD in undifferentiated RD cells (Figure 5A), and MSC occupancy diminished in RD cells that underwent RUNX1- (Figure 5B) or MD ~ E-mediated differentiation ( Additional file 11: Figure S7). High-throughput sequencing of the MyoD and MSC ChIP material (ChIP-seq) from undifferentiated RD cells with analysis restricted to sequences mapping to the miR-206 promoter revealed distinct MyoD and MSC peaks over two adjacent E-boxes (Figure 5C), indicating a MyoD bound E-box adjacent to a MSC bound E-box.

Electrophoretic mobility shift assaysdemonstrated that the E-box associated with the MyoD peakhad a higher affinity for MyoD compared to the E-box underthe MSC peak, but that both E-boxes can be bound by eitherMyoD or MSC in vitro (Additional file 12: Figure S8). Whenthe E-protein is in excess, gel shift assays show that MyoD:Eheterodimers can compete with MSC:E heterodimers to bindthe E-box under the MyoD ChIP-seq peak (Figure 5D, leftpanel, lane 1) and that decreasing the amount of MSC shiftsthe binding progressively toward MyoD:E protein heterodimers(lanes 2–5). Even with a relative excess of E-protein,MSC:E heterodimers outcompete MyoD:E heterodimers onthe E-box under the MSC ChIP-seq peak andMyoD:E bindingto this site occurred only when MSC levels were decreased (Figure 5D, right panel, lanes 1–5). Therefore the relative affinitiesof MSC:E and MyoD:E heterodimers for the two E-boxesin the miR-206 regulatory regions likely account for theobserved endogenous binding (Figure 5C) and suggest that decreasinglevels of MSC would result in occupancy of both E-boxes by MyoD.

Co-transfection experiments showed that MyoD:E12 heterodimers robustly activated the miR-206 reporter containing both E-boxes and this was prevented by MSC (Figure 5E, black bars). MSC also repressed activation by the MD ~ E dimer, suggesting the effect of MSC is due to DNA binding, not interference with the formation of MyoD:E dimers ( Additional file 13: Figure S9). Mutation of the MSC binding site made the reporter insensitive to activation by MyoD and E12 (Figure 5E, grey bars). This suggests that MSC is repressing miR-206 by physically occluding an E-box that MyoD needs to occupy for full activation. The MyoD and MSC ChIP-seq data also supports this conclusion. Compared to the MyoD peak in RD cells, there is a broadening of the MyoD peak in myotubes that appears to widen to include E-boxes located more proximally to the start of the miR-206 transcript (Figure 5C, bottom panel), suggesting that in differentiated muscle, MyoD occupies additional positions. Consistent with a model in which miR-206 activity requires multiple E-boxes to be bound by MyoD for full activation, mutation of the MyoD-binding E-box also resulted in a dramatic reduction in the ability of the reporter to be activated by MyoD and E12 ( Additional file 14: Figure S10).

### An alternative splice form of E2A modulates MyoD activation of miR-206

We previously described a developmentally regulated splice form of E2A that removes a portion of the first activation domain by splicing exon 2 directly to exon 5 that we called E2A-2/5. To determine whether the E2A-2/5 splice form contributed to the bHLH balance at the miR-206 promoter, we tested the response of RD cells to expression of a forced protein dimer consisting of MyoD and the E12-2/5 splice form of E12 (MD ~ E2/5). MD ~ E2/5 expressing cells formed substantially fewer myotubes ( Additional file 15: Figure S11A) while expressing roughly equivalent amounts of dimer ( Additional file 15: Figure S11B) compared to cells expressing the full-length MD ~ E dimer. MD ~ E expressing cells expressed substantially more *CKM* (Figure 5F), *RUNX1* (Figure 5G), and miR-206 (Figure 5H) than MD ~ E2/5 cells. However, in all cases, MD ~ E2/5 expressing cells still expressed more of the MyoD targets than control cells, suggesting that, while the specific E-protein partner is important for full activation of miR-206, the inhibitory effect of MSC is crucial.

## Discussion

We have previously proposed that RMS represent an arrested phase of normal development at the transition point between regulative growth and terminal differentiation, a transition regulated in part through the balance of repressive and activating bHLH protein dimers [8]. We showed that increasing the abundance of MyoD:E-protein heterodimers tipped the balance to differentiation and proposed that this heterodimer might target an unknown central integrating function since multiple myogenic repressors were down-regulated. One prediction of this earlier model was that modulating the abundance of any of the factors that impinge on the integrating function might be sufficient to induce differentiation in RMS. Our present study supports and extends this model by demonstrating that miR-206 integrates the activity of multiple proliferative and myogenic factors and acts as a switch that transitions the RMS from growth to differentiation.

To test whether different myogenic co-factors can induce differentiation in RMS, we chose one transcriptional activator and one repressor, RUNX1 and ZNF238, respectively. RUNX1 enhances MyoD activation at a variety of MyoD targets, including *ZNF238* and possibly *MYOG* and the *MEF* genes. In contrast, ZNF238 down-regulates multiple members of the inhibitory HES and HEY protein family and factors that drive proliferation. Our data on *ZNF238* regulation, motif analysis, and gene targets in this and previous work [11] suggest that the induction of this inhibitory factor serves two purposes: (1) to down-regulate genes that inhibit myogenesis; and (2) to interfere with MyoD binding at genes it might regulate in myoblasts. Despite differences in their direct targets, RUNX1 and ZNF238 both increase miR-206 transcription and lead to a terminally differentiated state. For RUNX1 we demonstrated a direct binding of the miR-206 promoter, whereas the mechanism for ZNF238 remains speculative, possibly through its suppression of *ID* gene expression, which would increase the proportion of productive MyoD:E-protein heterodimers. While *in vitro* in nature, our data on the relative ability of MyoD:E and MSC:E to shift the regulatory E-box sequences that control miR-206 expression (Figure 5D and Additional file 12: Figure S8) suggest that even relatively small changes in the availability of E-protein partners could make a dramatic difference in the expression of miR-206.

*MSC*, a bHLH that inhibits myogenesis [32], suppresses the activation of miR-206 by binding an E-box required for induction by MyoD. A requirement for more than one MyoD-bound E-box to drive full target activation has been described before [33-37], and it is currently unclear whether MSC is simply preventing MyoD binding or recruiting repressive factors to the locus. The fact that the miR-206 locus has acetylated H4 even when not robustly expressed suggests that MSC may have a simple obstructionist role at this locus. Future work will be necessary to determine the relative roles of MSC and MyoD at miR-206 and other myogenic targets.

**Figure 6:**
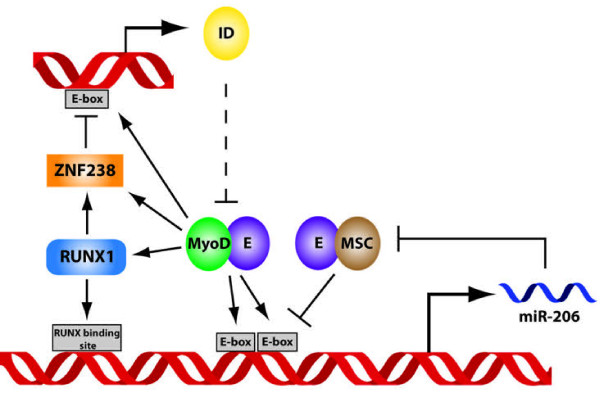
**miR-206 integrates the output of oscillating circuits and acts as a genetic switch to transition from growth to differentiation.** The experimental data support a network model composed of coupled oscillators with miR-206 functioning as a switch regulating the transition from one stable state to another. In myoblasts, MyoD, E-proteins, and ID proteins compose the first oscillating circuit: (1) MyoD:E heterodimers bind an E-box in the regulatory regions of the ID2 and ID3 genes and drive ID transcription; (2) the ID protein competitively forms dimers with the E-protein, limiting the production of active MyoD:E-protein heterodimers; (3) the decline in active MyoD:E-heterodimers results in decreased ID production; and (4) the decreased ID permits an increase in active MyoD:E-protein heterodimers and more ID production. The second oscillating circuit is composed of MyoD, E-proteins, MSC, and miR-206: (1) MyoD:E-protein and MSC:E-protein heterodimers compete for binding at the E-box in the miR-206 regulatory region, which oscillates between MyoD-activated and MSC-repressed states; (2) limiting amounts of E-protein prevent full activation by MyoD; and (3) low levels of miR-206 prevent full suppression by MSC. These circuits are coupled by their shared response to the concentration of active MyoD:E-protein heterodimers. The oscillating circuits bifurcate to a new determined state when the concentration and/or activity of the MyoD:E-proteins become sufficient to activate the expression of RUNX1 and ZNF238 in a feed-forward circuit that blocks the expression of the ID genes and permits the accumulation of active MyoD:E-protein complexes. The increase of MyoD:E-protein heterodimers together with RUNX produces higher miR-206 expression, and the increased miR-206 suppresses MSC and other inhibitors of differentiation.

Data reported in this manuscript, when combined with previous data from us and others [8,11,18,19] suggests a specific model for the regulation of miR-206 that involves nested positive and negative feed-forward and feed-back loops to create a molecular switch for regulating the transition from growth to differentiation in myogenic cells (Figure 6). In replicating myoblasts, a MyoD:E-protein heterodimer binds an E-box site in the regulatory regions of ID2 and ID3 [11] creating the potential for an oscillating circuit: any increase in MyoD activity would increase ID expression, which would dampen MyoD activity by decreasing the availability of the E-protein dimer partner. However, if MyoD:E-protein heterodimers pass a threshold of activity sufficient to initiate a feed-forward circuit activating RUNX1 and ZNF238, then ZNF238 shuts off ID production by occluding the MyoD binding sites and thereby relieving the negative-feedback regulation of MyoD. The increased MyoD and RUNX1 activity can then more effectively compete with MSC on the miR-206 regulatory regions and the increased miR-206 levels feed back to inhibit MSC, and likely other growth promoting factors [8,19,20,24,38], thereby locking the cell into a committed differentiation program. Therefore, miR-206 integrates the output of oscillating circuits and acts as a genetic switch to transition the cell from a proliferative growth phase to differentiation.

Hematological malignancies are often categorized based on an arrested transition between stages of cellular differentiation. Our work suggests that the same might apply to RMS and possibly other solid tumors. bHLH factors control cell fate and differentiation in multiple cell types and a balance among bHLH dimer partners and other co-factors might establish similar ‘tipping points’ at critical genes that regulate the transition from regulative growth to differentiation. Our emerging model of multiple pathways (some functioning as oscillating circuits) integrated by switch-points for differentiation has significant implications for drug therapies to induce differentiation. Different cell types may not exhibit identical convergence of pathways. Therefore, combining multiple drugs that each has a small effect on different components might induce differentiation in the target cells while exhibiting low toxicity and few off-target effects.

## Conclusions

Our results in a rhabdomyosarcoma cell line provide evidence for feed-forward and feed-back regulatory circuits in myogenic cells that regulate miR-206 expression as a genetic switch to transition a muscle cell from growth to differentiation. One circuit is composed of MyoD, RUNX1, ZNF238, and ID2/3 and might function, at least in part, to regulate the availability of E-proteins to form heterodimers with MyoD; whereas the second circuit composed of MyoD, RUNX1, and MSC regulates the expression of miR-206. These have characteristics of coupled oscillatory circuits that can dampen the activity of MyoD during expansionary growth, or, under differentiation promoting conditions, can be switched off and thereby enhance MyoD activity and drive differentiation. Modulating the abundance of multiple different components of these coupled circuits can drive differentiation, suggesting that multiple targets within these circuits might be candidates for targeting differentiation-inducing therapeutics.

## Abbreviations

bHLH, Basic helix-loop-helix; ChIP, Chromatin immunoprecipitation; ChIP-seq, Chromatin immunoprecipitation coupled to high-throughput sequencing; RMS, Rhabdomyosarcoma.

## Competing interests

The authors declare no potential conflicts of interest.

## Authors’ contributions

KLM contributed to all experimental designs, performed all experiments not otherwise mentioned below, and drafted the manuscript. ZY performed the ChIP-seq analysis and contributed to the gene expression and GO analyses. JMY analyzed the gene expression data and performed the GO analysis. YC performed the miRNA array analysis. SJT conceived of the project, contributed to all experimental designs, and edited the manuscript. All authors read and approved the final manuscript.

## Supplementary Material

Additional file 1 **Table S1.**Primer and oligonucleotide sequences. Click here for file

Additional file 2 **Figure S1.**RD cells infected with ZNF238 and RUNX1 viruses increase expression of the appropriate factor. (**A**) RT-PCR for *ZNF238* in RD cells infected with either a control virus or the ZNF238-containing virus. *TIMM17b* is used as a loading control. (**B**) Western blot using whole cell lysates for RUNX1 in control and RUNX1 virus infected RD cells. The blot was then stripped and reprobed for alpha-tubulin as a loading control. Bands were confirmed to be of the correct size through a protein size ladder (not shown). Click here for file

Additional file 3 **Figure S2.**RUNX1 differentiates alveolar subtype RMS cells. (**A**) Western blots on whole cell lysates from RhJT cells infected with either a RUNX1-expressing or control virus. MHC is myosin heavy chain, a marker of myogenesis, and alpha-tubulin is the loading control. Blots were serially stripped and reprobed, and bands confirmed to be of the correct size. (**B**) RT-PCR for *CKM* (muscle specific creatine kinase) on cells treated as in A. *TIMM17b* is the internal control. Click here for file

Additional file 4 **Table S2.**miRNA changes in response to MD~E expression in RD cells. Click here for file

Additional file 5 **Figure S3.**Differential effects on miRNA expression by the forced MD~E dimer. (**A**) miRNA northern blot for miR-199a* (also known as miR-199a-5p) from RD cells either transduced with a control or MD~E virus. (**B**) miRNA northern blot for miR-29b as in panel A. Click here for file

Additional file 6 **Figure S4.**miR-206 affects alveolar subtype RMS cells and miR-133b does not share its effects**.** (**A**) Immunostains for MHC in RhJT cells transfected with either a pre-miR-206 or control construct. DAPI stains all nuclei. (**B**) Stains as in A after transfection of RD cells with pre-miR-133b or a control construct. (**C**) qPCR for *CKM* in RD cells treated as in B. qPCR data was normalized to *TIMM17b* and control set to 1, with bars representing the mean ± SEM of three independent experiments. *: *P*<0.05. Click here for file

Additional file 7 **Figure S5.**MyoD activity increases *ZNF238* expression during myogenic conversion. 10T1/2 fibroblast cells expressing an estradiol-inducible version of MyoD were induced to undergo myogenesis by addition of beta-estradiol to the culture medium. RNA was taken at the indicated times under indicated conditions and qPCR performed to quantitate the relative levels of *ZNF238*. All bars indicate the mean ± SEM of at least three independent experiments. Time 0 was set to 1, and *TIMM17b* served as the internal control. *: *P*<0.05. Click here for file

Additional file 8 **Table S3.**Top GO categories of genes regulated by RUNX1, ZNF238, and miR-206. Click here for file

Additional file 9 **Table S4.**Select potential regulators of myogenesis affected by RUNX1, ZNF238, and miR-206. Click here for file

Additional file 10 **Figure S6.**The miR-206 promoter in RD cells is bound by MyoD and has acetylated histone H4. (**A**) ChIP for MyoD in RD cells in differentiation media shows MyoD enrichment upstream of miR-206, compared to a control locus at a non-expressed gene (hemoglobin beta). (**B**) Site-specific ChIPs in RD cells for acetylated histone H4, at hemoglobin beta (control), miR-206, and the myogenin promoter (MYOG). ChIP results are represented as the mean ± SEM of at least three independent experiments. Relative enrichment is calculated as the ratio of the % of input amplified with antibody to the % of input amplified with no antibody. *: *P*<0.05; **: *P*<0.01. Click here for file

Additional file 11 **Figure S7.**MSC occupancy in the miR-206 promoter diminishes with MD~E differentiation. ChIP for MSC in the miR-206 promoter in RD cells either transduced with empty virus (Control), or differentiated through the expression of the forced MD~E protein dimer (MD~E). Values are the means ± standard deviation of two independent experiments. Corrected relative enrichment equals the relative enrichment at miR-206/the relative enrichment at the control locus. Relative enrichment is calculated as the ratio of the % of input amplified with antibody to the % of input amplified with no antibody. Click here for file

Additional file 12 **Figure S8.***In vitro* assessment of MyoD and MSC binding in the miR-206 promoter. Electrophoretic mobility shift assays were performed using *in vitro* translated proteins as indicated and probes that represent the DNA sequence under either the E-box occupied most prominently by MyoD in RD cells as assessed by ChIP-seq results (MyoD-binding E-box) or the E-box occupied most prominently by MSC (MSC-binding E-box). Bound complexes were competed with cold competitor probes prepared at the indicated excess. Click here for file

Additional file 13 **Figure S9.**MSC inhibits the activation of the miR-206 reporter by the forced MD~E dimer. Luciferase assay results in RD cells using the miR-206 promoter reporter with constant amounts of MyoD and E12 introduced individually or as the forced dimer, in the presence of varying amounts of co-transfected MSC. - indicates no MSC was added, 1x indicates that the MSC transfected was equal by mass to the amount of MyoD or MD~E, and 0.1x indicates that the MSC transfected was equal to 1/10th that amount. Results are indicated as the means ± SEM from three independent experiments. Control indicates the results from transfection with empty vector. Click here for file

Additional file 14 **Figure S10.**Strong miR-206 activation is dependent on multiple E-boxes. Luciferase assay results in RD cells with transient transfection as indicated using the miR-206 promoter and a reporter in which the E-box exhibiting the peak of MyoD occupancy in RD cells (indicated by the red marker in Figure 5C) has been mutated and eliminated as a site of bHLH binding. Results are indicated as the means ± SEM from three independent experiments. Control indicates the results from transfection with empty vector. *: *P*<0.05. Click here for file

Additional file 15 **Figure S11.**MD~E expression results in greater myotube formation than MD~E2/5 expression. (**A**) Light microscopy images of RD cells transduced with either control virus (Control) or virus expressing either the MD~E or MD~E2/5 forced protein dimers and allowed to differentiate for 24 h. Arrows indicate representative cells that have appeared to form myotubes. (**B**) Western blot for MyoD and alpha-tubulin, as a loading control, from cells treated as in (A). The size of the bands detected with the MyoD antibody in MD~E and MD~E2/5 lanes are as expected given the approximate calculated size of the forced dimers. Click here for file
