# Supplementary material for: miR-206 integrates multiple components of differentiation pathways to control the transition from growth to differentiation in rhabdomyosarcoma cells
Source: Skelet Muscle. 2012 Apr 29;2:7. doi: 10.1186/2044-5040-2-7 (PMC3417070; doi:10.1186/2044-5040-2-7)
Supplement: Additional file 1 — Table S1.Primer and oligonucleotide sequences. [file 2044-5040-2-7-S1.doc]

**Supplemental Table S1. Primer and oligonucleotide sequences.**

| **Primer/Probe Name** | **Forward primer or probe sequence** | **Reverse primer or probe sequence** |
| --- | --- | --- |
| *RT-PCR and qRT-PCR* |  |  |
| CKM | ccaagttcgaggagatcctc | agctgcacctgttctacttcg |
| TIMM17b | ggagccttcactatgggtgt | cacagcattggcactacctc |
| Timm17b (mouse) | tgtcattggtggtggagtct | actgcaaagcttcctccaat |
| RUNX1 (set 1) | gggaactgtcaagctggtgt | gcctgctctcctgtgctatc |
| RUNX1 (set 2) | tttccagtcgactctcaacg | gtcggggagtaggtgaagg |
| ZNF238 (isoform 1) | gcaggactcagaggaaagga | ctctgctcgctcagacactg |
| ZNF238 (isoform 2) | gagcgctgaaaagttgttcc | ctctgctcgctcagacactg |
| ZNF238 (mouse) | tccccagtgagagaaatctga | tgctgaagctgcaatatcctt |
| ID2 | cccagaacaagaaggtgagc | atagtgggatgcgagtccag |
| ID3 | ctggacgacatgaaccactg | gtagtcgatgacgcgctgta |
| MYOG | ggccacagatgccactactt | gctttacctccctggaaagg |
| MEF2D | ctctttgccgtgacaacacc | ctcatgaacggtctgggaac |
| MYCN | cacaaggccctcagtacctc | cacagtgaccacgtcgattt |
| E2F2 | ctacacaccgctgtacccg | ccagatccagcttccttttg |
| RCOR2 | tcagctcatctccctcaagc | tagtggatcaataccgccct |
| HEYL | atcgacgtgggccaagag | atccctctgcgtttcttcct |
| HEY1 | tggatcacctgaaaatgctg | cgaaatcccaaactccgata |
| Pri-miR-206 (RT-PCR) | gtttcggcaagtgcctcct | ctcttgcttccttggtgagg |
| Pri-miR-206 (qPCR) | tgctgtgagtgaggttcagg | cagggttgtggtgtgaagtg |
| NOTCH | tgtgcaaatggaggtcgtt | cctgagtgacaggggtcct |
| DLL3 | catcgaaacctggagagagg | cctgcgcgctgaatgtc |
| *microRNA Northerns* |  |  |
| miR-206 | ccacacacttccttacattcca | N/A |
| miR-133b | tagctggttgaaggggaccaaa | N/A |
| miR-16 | cgccaatatttacgtgctgcta | N/A |
| miR-29b | aacactgatttcaaatggtgcta | N/A |
| miR-199a* | taaccaatgtgcagactactgt | N/A |
| *EMSA* |  |  |
| MSC-bound E-box | tggatgggcagctgctgcccat | atgggcagcagctgcccatcca |
| MyoD-bound E-box | tggctcaacagctgccaatgtc | gacattggcagctgttgagcca |
| *ChIP qPCR* |  |  |
| miR-206 | caacaagcacccaaaacaga | ttccacattcacgcagagag |
| HBB (hemoglobin beta control locus) | aacggcagacttctcctcagg | agtcagggcagagccatcta |
| miR-206 (RUNX binding) | tggcatatgtttccccattt | gttgagccactcagggtctg |
| ZNF238 | ccacagtcagctggatcaga | gagggcagctcacaaggtag |
| MYH8 enhancer (RUNX control locus | tgtggctatctctgtgtgcag | ttagattttgggggatggtg |
| *Luciferase cloning* |  |  |
| miR-206 | gaatgctagcctgtccttgattttaccc | caatagatctttgtgcagctacagtcta |
