# Supplementary figures and images for: miR-206 integrates multiple components of differentiation pathways to control the transition from growth to differentiation in rhabdomyosarcoma cells
Source: Skelet Muscle. 2012 Apr 29;2:7. doi: 10.1186/2044-5040-2-7 (PMC3417070; doi:10.1186/2044-5040-2-7)

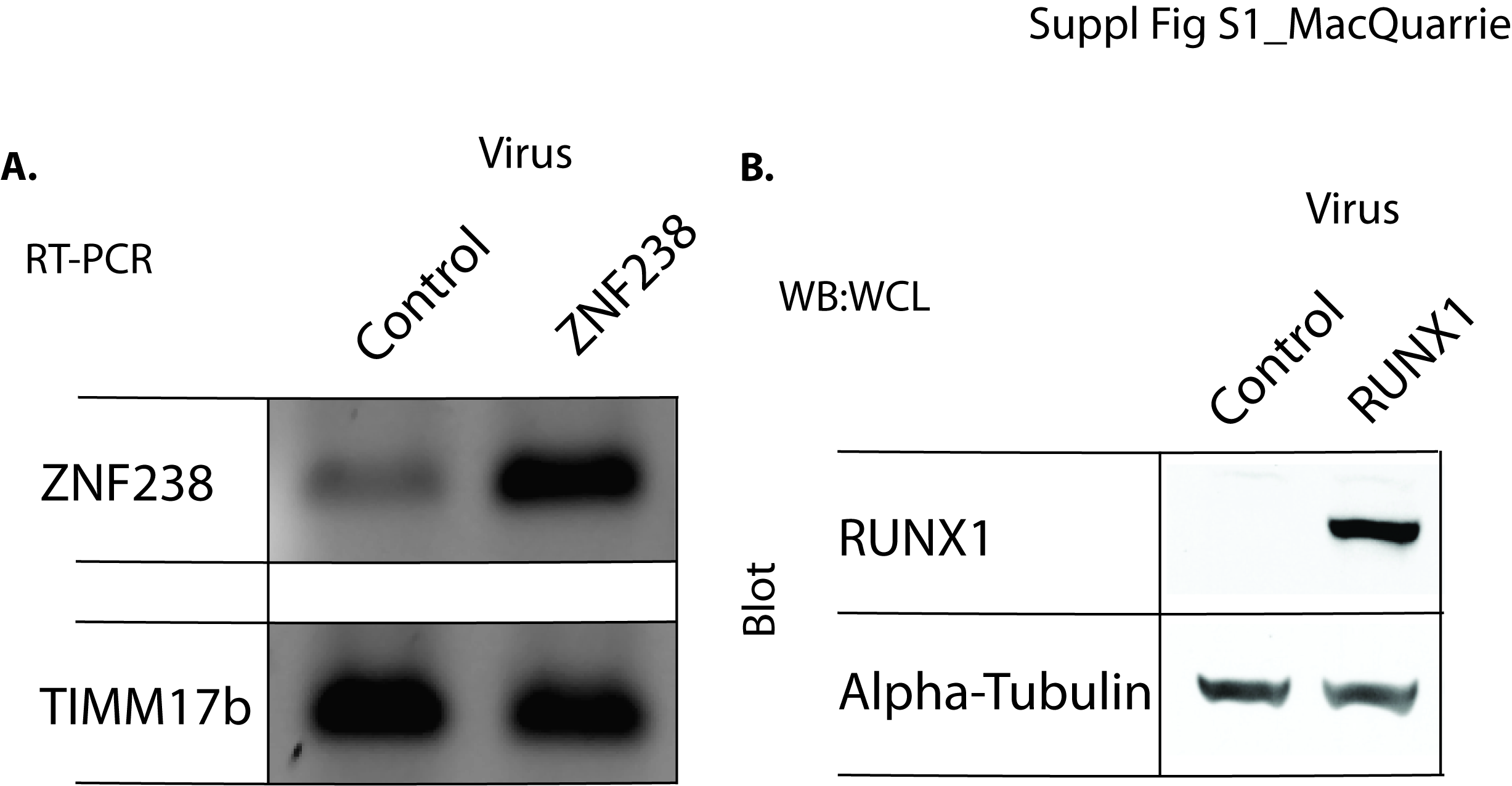

Supplement: Additional file 2 — Figure S1.RD cells infected with ZNF238 and RUNX1 viruses increase expression of the appropriate factor. (A) RT-PCR for ZNF238 in RD cells infected with either a control virus or the ZNF238-containing virus. TIMM17b is used as a loading control. (B) Western blot using whole cell lysates for RUNX1 in control and RUNX1 virus infected RD cells. The blot was then stripped and reprobed for alpha-tubulin as a loading control. Bands were confirmed to be of the correct size through a protein size ladder (not shown). [file 2044-5040-2-7-S2.tiff]

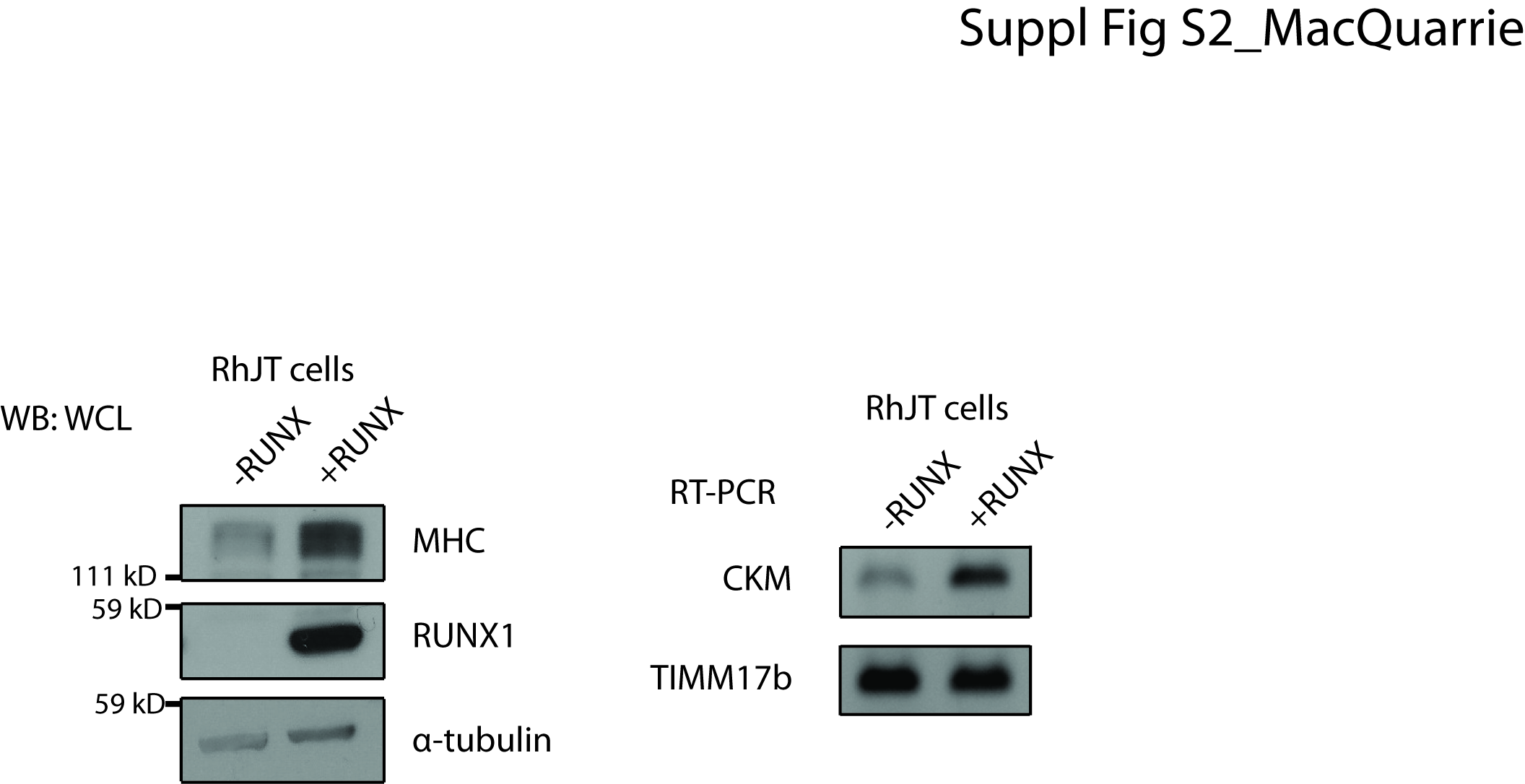

Supplement: Additional file 3 — Figure S2.RUNX1 differentiates alveolar subtype RMS cells. (A) Western blots on whole cell lysates from RhJT cells infected with either a RUNX1-expressing or control virus. MHC is myosin heavy chain, a marker of myogenesis, and alpha-tubulin is the loading control. Blots were serially stripped and reprobed, and bands confirmed to be of the correct size. (B) RT-PCR for CKM (muscle specific creatine kinase) on cells treated as in A. TIMM17b is the internal control. [file 2044-5040-2-7-S3.tiff]

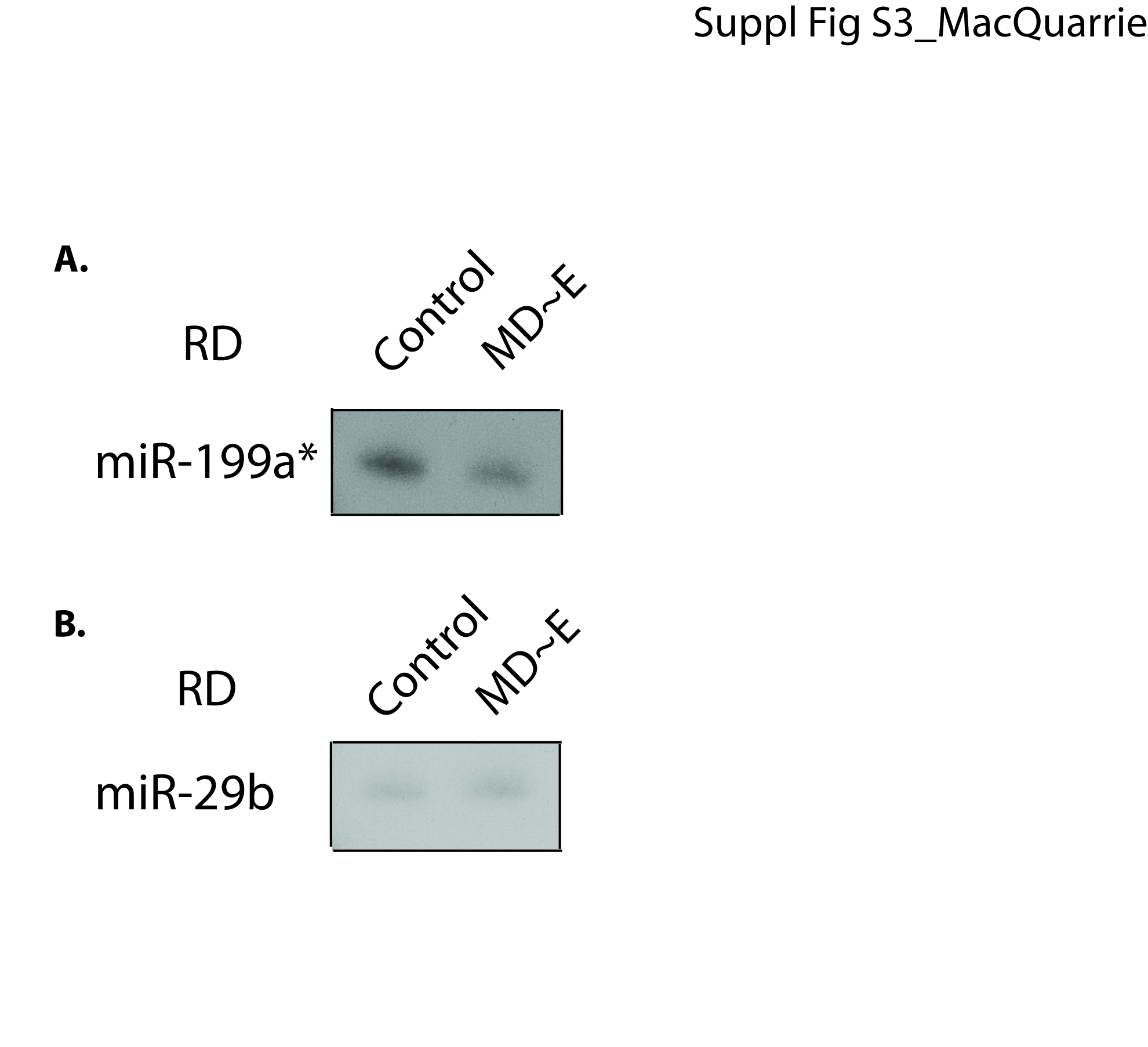

Supplement: Additional file 5 — Figure S3.Differential effects on miRNA expression by the forced MD~E dimer. (A) miRNA northern blot for miR-199a* (also known as miR-199a-5p) from RD cells either transduced with a control or MD~E virus. (B) miRNA northern blot for miR-29b as in panel A. [file 2044-5040-2-7-S5.tiff]

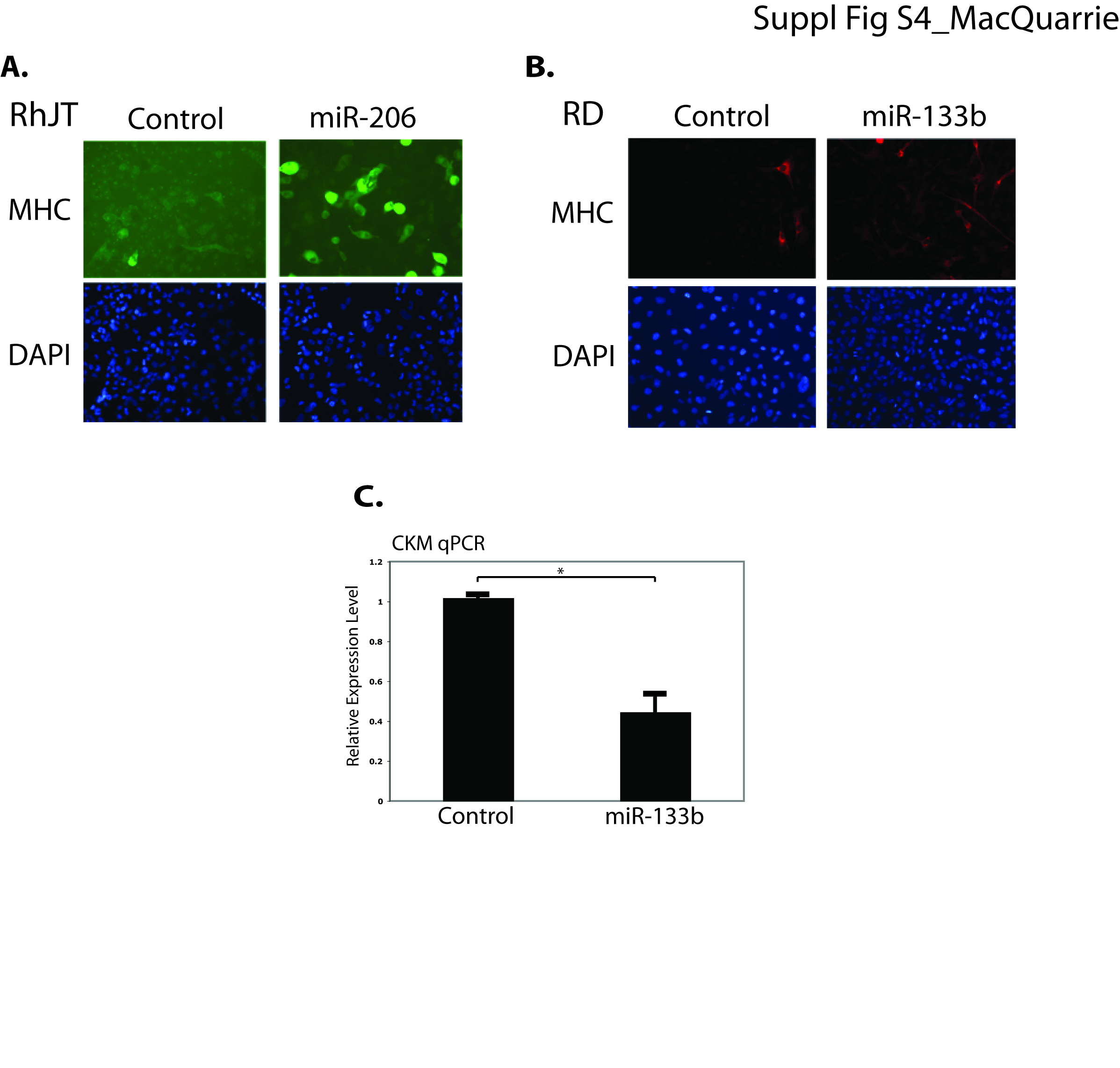

Supplement: Additional file 6 — Figure S4.miR-206 affects alveolar subtype RMS cells and miR-133b does not share its effects. (A) Immunostains for MHC in RhJT cells transfected with either a pre-miR-206 or control construct. DAPI stains all nuclei. (B) Stains as in A after transfection of RD cells with pre-miR-133b or a control construct. (C) qPCR for CKM in RD cells treated as in B. qPCR data was normalized to TIMM17b and control set to 1, with bars representing the mean ± SEM of three independent experiments. *: P<0.05. [file 2044-5040-2-7-S6.tiff]

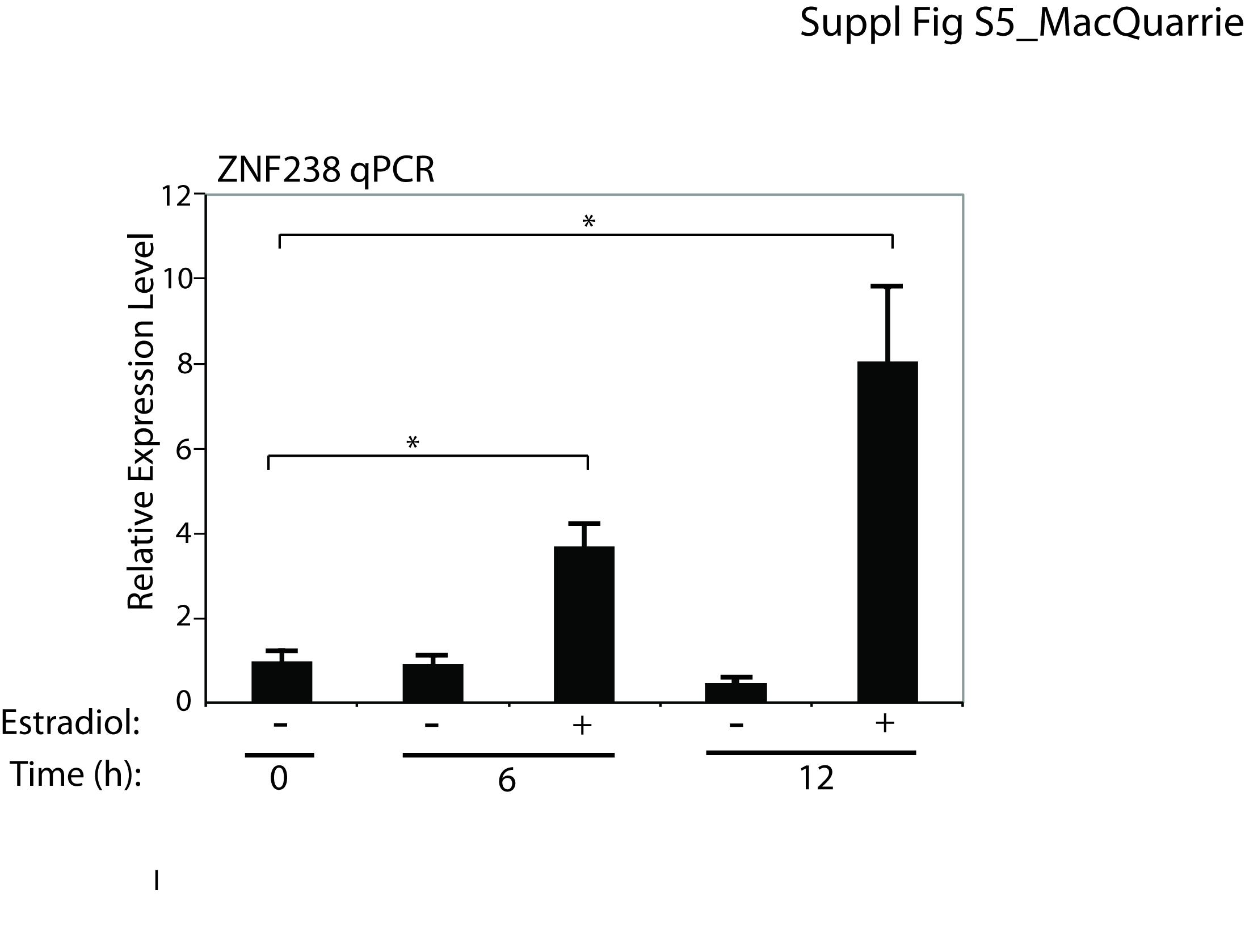

Supplement: Additional file 7 — Figure S5.MyoD activity increases ZNF238 expression during myogenic conversion. 10T1/2 fibroblast cells expressing an estradiol-inducible version of MyoD were induced to undergo myogenesis by addition of beta-estradiol to the culture medium. RNA was taken at the indicated times under indicated conditions and qPCR performed to quantitate the relative levels of ZNF238. All bars indicate the mean ± SEM of at least three independent experiments. Time 0 was set to 1, and TIMM17b served as the internal control. *: P<0.05. [file 2044-5040-2-7-S7.tiff]

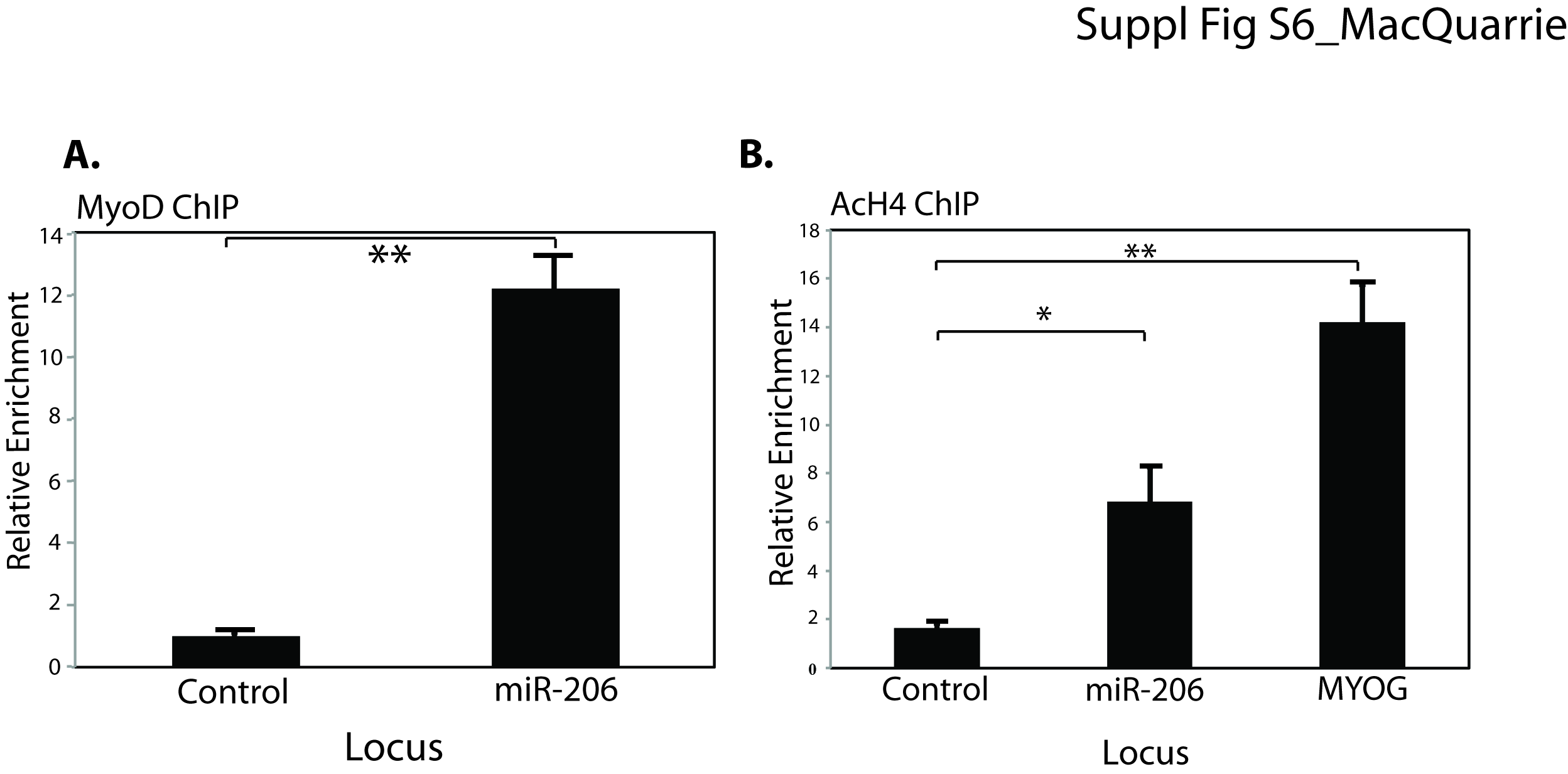

Supplement: Additional file 10 — Figure S6.The miR-206 promoter in RD cells is bound by MyoD and has acetylated histone H4. (A) ChIP for MyoD in RD cells in differentiation media shows MyoD enrichment upstream of miR-206, compared to a control locus at a non-expressed gene (hemoglobin beta). (B) Site-specific ChIPs in RD cells for acetylated histone H4, at hemoglobin beta (control), miR-206, and the myogenin promoter (MYOG). ChIP results are represented as the mean ± SEM of at least three independent experiments. Relative enrichment is calculated as the ratio of the % of input amplified with antibody to the % of input amplified with no antibody. *: P<0.05; **: P<0.01. [file 2044-5040-2-7-S10.tiff]

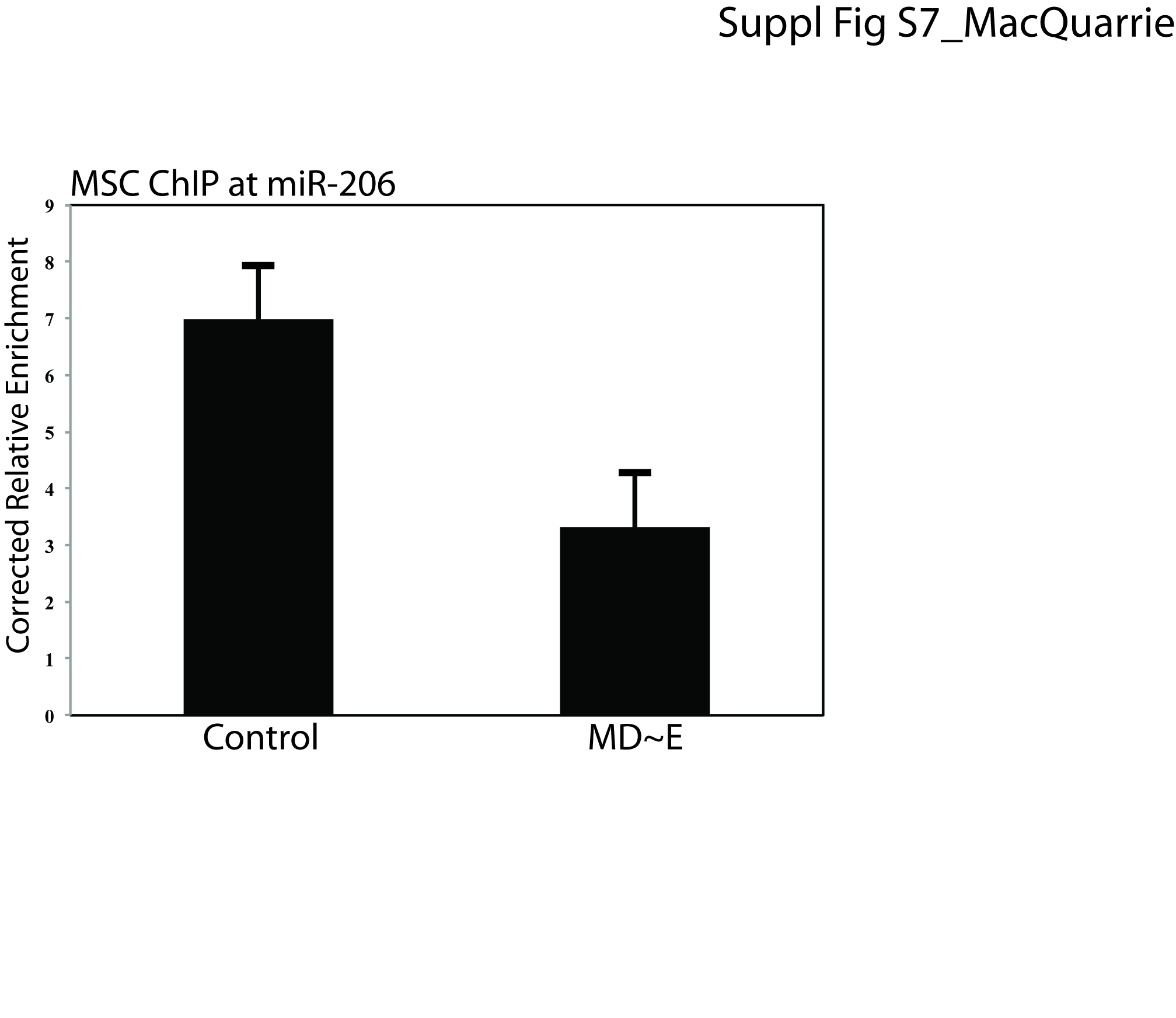

Supplement: Additional file 11 — Figure S7.MSC occupancy in the miR-206 promoter diminishes with MD~E differentiation. ChIP for MSC in the miR-206 promoter in RD cells either transduced with empty virus (Control), or differentiated through the expression of the forced MD~E protein dimer (MD~E). Values are the means ± standard deviation of two independent experiments. Corrected relative enrichment equals the relative enrichment at miR-206/the relative enrichment at the control locus. Relative enrichment is calculated as the ratio of the % of input amplified with antibody to the % of input amplified with no antibody. [file 2044-5040-2-7-S11.tiff]

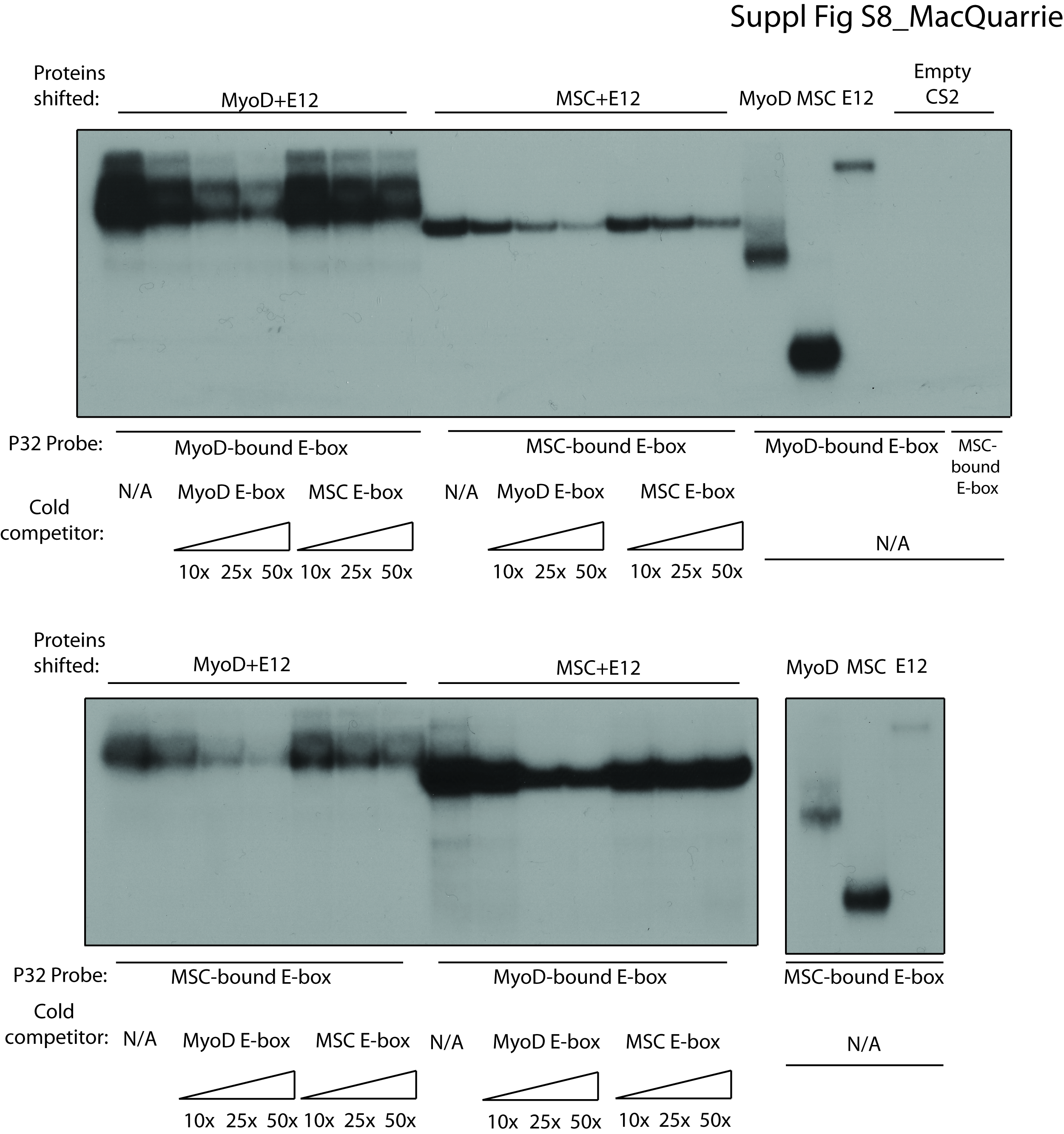

Supplement: Additional file 12 — Figure S8.In vitro assessment of MyoD and MSC binding in the miR-206 promoter. Electrophoretic mobility shift assays were performed using in vitro translated proteins as indicated and probes that represent the DNA sequence under either the E-box occupied most prominently by MyoD in RD cells as assessed by ChIP-seq results (MyoD-binding E-box) or the E-box occupied most prominently by MSC (MSC-binding E-box). Bound complexes were competed with cold competitor probes prepared at the indicated excess. [file 2044-5040-2-7-S12.tiff]

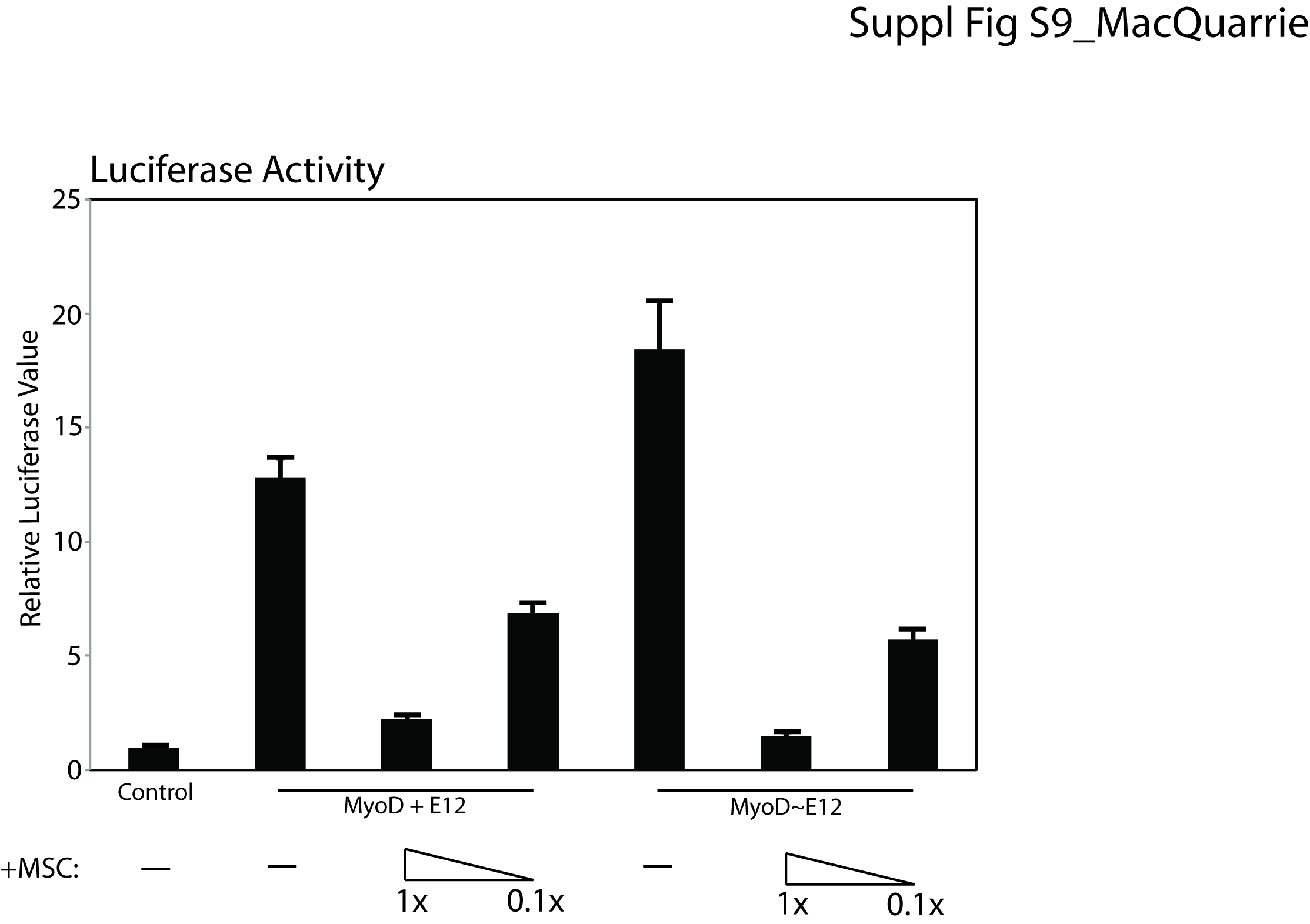

Supplement: Additional file 13 — Figure S9.MSC inhibits the activation of the miR-206 reporter by the forced MD~E dimer. Luciferase assay results in RD cells using the miR-206 promoter reporter with constant amounts of MyoD and E12 introduced individually or as the forced dimer, in the presence of varying amounts of co-transfected MSC. - indicates no MSC was added, 1x indicates that the MSC transfected was equal by mass to the amount of MyoD or MD~E, and 0.1x indicates that the MSC transfected was equal to 1/10th that amount. Results are indicated as the means ± SEM from three independent experiments. Control indicates the results from transfection with empty vector. [file 2044-5040-2-7-S13.tiff]

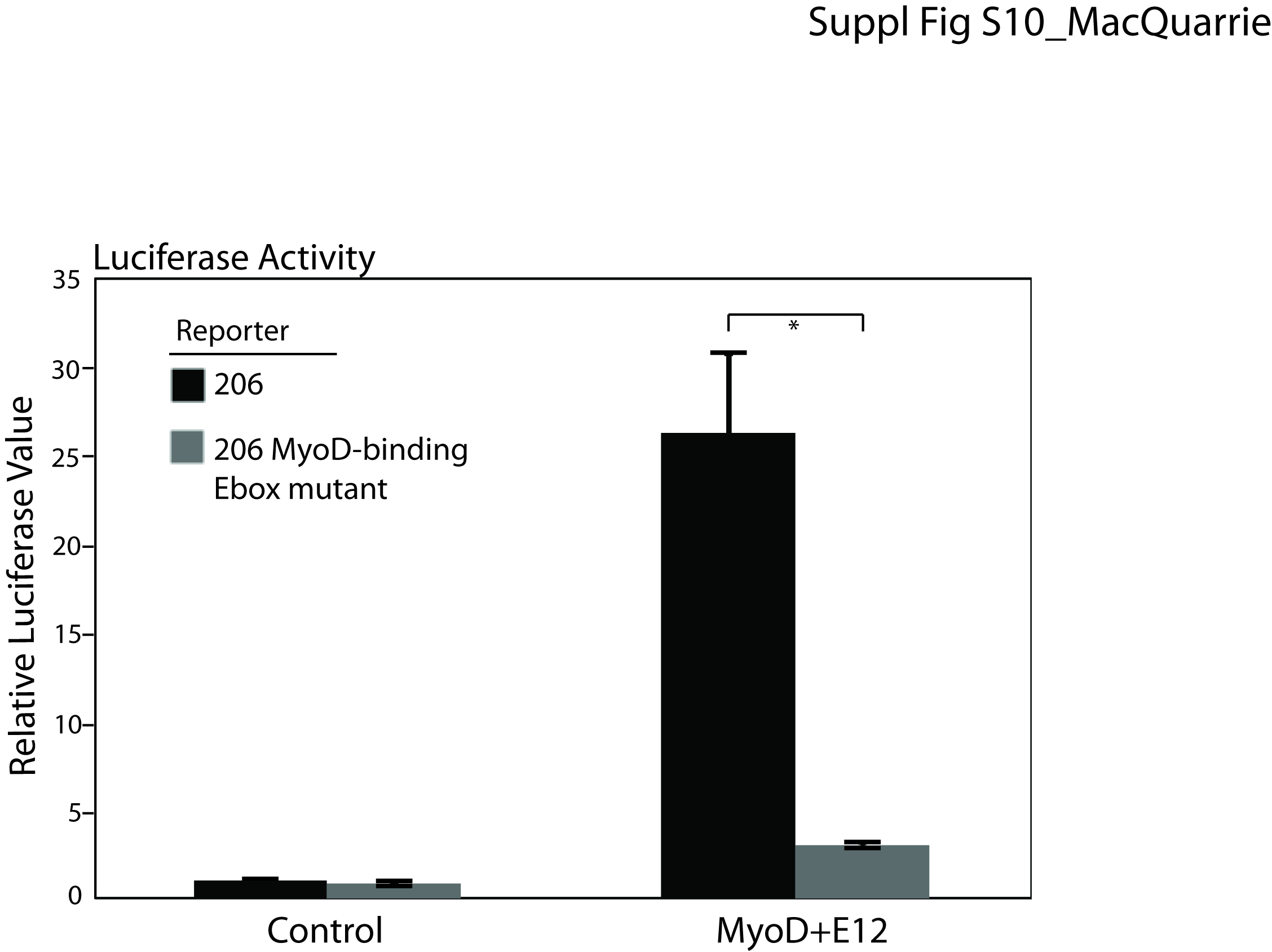

Supplement: Additional file 14 — Figure S10.Strong miR-206 activation is dependent on multiple E-boxes. Luciferase assay results in RD cells with transient transfection as indicated using the miR-206 promoter and a reporter in which the E-box exhibiting the peak of MyoD occupancy in RD cells (indicated by the red marker in Figure 5C) has been mutated and eliminated as a site of bHLH binding. Results are indicated as the means ± SEM from three independent experiments. Control indicates the results from transfection with empty vector. *: P<0.05. [file 2044-5040-2-7-S14.tiff]

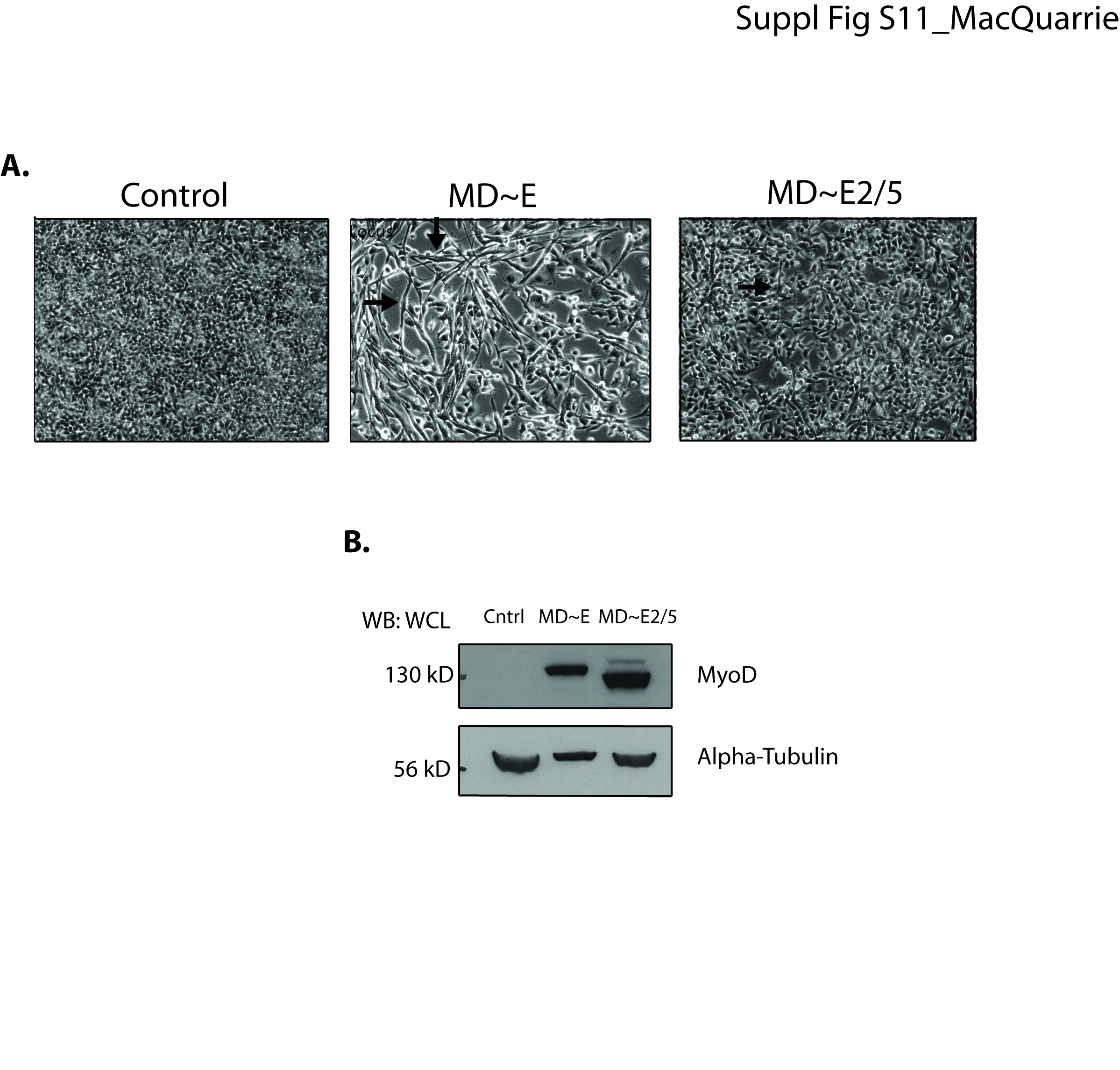

Supplement: Additional file 15 — Figure S11.MD~E expression results in greater myotube formation than MD~E2/5 expression. (A) Light microscopy images of RD cells transduced with either control virus (Control) or virus expressing either the MD~E or MD~E2/5 forced protein dimers and allowed to differentiate for 24 h. Arrows indicate representative cells that have appeared to form myotubes. (B) Western blot for MyoD and alpha-tubulin, as a loading control, from cells treated as in (A). The size of the bands detected with the MyoD antibody in MD~E and MD~E2/5 lanes are as expected given the approximate calculated size of the forced dimers. [file 2044-5040-2-7-S15.tiff]
