# Supplementary material for: miR-206 integrates multiple components of differentiation pathways to control the transition from growth to differentiation in rhabdomyosarcoma cells
Source: Skelet Muscle. 2012 Apr 29;2:7. doi: 10.1186/2044-5040-2-7 (PMC3417070; doi:10.1186/2044-5040-2-7)
Supplement: Additional file 4 — Table S2.miRNA changes in response to MD~E expression in RD cells. [file 2044-5040-2-7-S4.doc]

**Supplemental Table S2.** miRNA changes in response to MD~E expression in RD cells.

| **Replicate** | **miRNA ID§** | **FC (log_2_)*** |
| --- | --- | --- |
|  |  |  |
| **Biological Replicate #1** | **hsa-miR-206, mmu-miR-206, rno-miR-206, dre-miR-206, gga-miR-206, mdo-miR-206, mne-miR-206, ppy-miR-206, xtr-miR-206** | 1.345; 1.3225 |
|  | hsa-miR-663 | 1.103 |
|  | mml-miR-133a, ppy-miR-133a | 1.015; 1.0385; 1.0005 |
|  | hsa-miR-133a-133b | 1.032; 1.0155 |
|  | **cel-miR-243** | 1.0215 |
|  | **hsa-miR-199a*, mmu-miR-199a*, bta-miR-199a*, dre-miR-199*, gga-miR-199*, xtr-miR-199a*** | -1.015 |
|  | hsa-miR-143 | -1.025 |
| **Biological Replicate #2** | **hsa-miR-206, mmu-miR-206, rno-miR-206, dre-miR-206, gga-miR-206, mdo-miR-206, mne-miR-206, ppy-miR-206, xtr-miR-206** | 1.081; 1.021 |
|  | hsa-miR-125a, mmu-miR-125a, rno-miR-125a, bta-miR-125a | -1.004 |
|  | hsa-miR-335, mmu-miR-335, rno-miR-335 | -1.021 |
|  | mghv-miR-M1-6 | -1.025 |
|  | hsa-miR-196b, mmu-miR-196b, rno-miR-196b, mdo-miR-196b | -1.0305 |
|  | ptc-miR478a-ptc-miR478b-ptc-miR478c | -1.0325 |
|  | dme-miR-6,dps-mir-6 | -1.0865 |
|  | **hsa-miR-199a*, mmu-miR-199a*, bta-miR-199a*, dre-miR-199*, gga-miR-199*, xtr-miR-199a*** | -1.106 |
|  | hsa-miR-653 | -1.11 |
|  | hsa-miR-99b, mmu-miR-99b, rno-miR-99b | -1.1225; -1.137 |
|  | ath-miR160a-, ath-miR160b-, ath-miR160c, gma-miR160, mtr-miR160, osa-miR160a-, osa-miR160b-, osa-miR160c-, osa-miR160d, ptc-miR160a-, ptc-miR160b-, ptc-miR160c-, ptc-miR160d, sbi-miR160d-, sbi-miR160a-, sbi-miR160c-, sbi-miR160b-, sbi-miR160e, zma-miR160a-, zma-miR160c-, zma-miR160d-, zma-miR160b-, zma-miR160e | -1.182 |
|  | cbr-miR-249 | -1.2025 |
|  | osa-miR169f-, osa-miR169g, ptc-miR169r, sbi-miR169c-, sbi-miR169d, zma-miR169f-, zma-miR169g-, zma-miR169h | -1.436 |
| **Biological Replicate #1; Experimental Repeat #2** | hsa-miR-302a, mmu-miR-302 | 1.3945 |
|  | osa-miR164c | 1.2045 |
|  | **hsa-miR-206, mmu-miR-206, rno-miR-206, dre-miR-206, gga-miR-206, mdo-miR-206, mne-miR-206, ppy-miR-206, xtr-miR-206** | 1.0395 |
|  | hsa-miR-204, mmu-miR-204, rno-miR-204, dre-miR-204, fru-miR-204, gga-miR-204-211, ggo-miR-204, mdo-miR-204, mne-miR-204, ppa-miR-204, ppy-miR-204, ptr-miR-204, sla-miR-204, ssc-miR-204, tni-miR-204a, xtr-miR-204 | -1.0145 |
|  | hsa-miR-548b | -1.016 |
|  | mmu-miR-684 | -1.025 |
|  | hsa-miR-376b | -1.045 |
|  | mmu-miR-505 | -1.0675 |
|  | hsa-miR-214, mmu-miR-214, rno-miR-214, age-miR-214, bta-miR-214, dre-miR-214, fru-miR-214, ggo-miR-214, mdo-miR-214, mml-miR-214, mne-miR-214, ppa-miR-214, ppy-miR-214, ptr-miR-214, sla-miR-214, ssc-miR-214, tni-miR-214, xtr-miR-214 | -1.088; -1.016 |
|  | ath-miR159a, gma-miR159, ptc-miR159a-, ptc-miR159b-, ptc-miR159c | -1.137 |
|  | mmu-miR-467a | -1.184 |
|  | cel-miR-84 | -1.2475 |
|  | hsa-miR-589 | -1.258 |
|  | dre-miR-27c, fru-miR-27c, tni-miR-27c | -1.4145 |
|  | hsa-miR-549 | -1.75 |
| **Biological Replicate #2; Experimental Repeat #2** | ath-miR394a-, ath-miR394b, osa-miR394, ptc-miR394a-, ptc-miR394b, sbi-miR394a-, sbi-miR394b, zma-miR394a-, zma-miR394b | 1.801 |
|  | aga-miR-9c, dme-miR-9c, dps-miR-9c | 1.252 |
|  | **hsa-miR-206, mmu-miR-206, rno-miR-206, dre-miR-206, gga-miR-206, mdo-miR-206, mne-miR-206, ppy-miR-206, xtr-miR-206** | 1.247; 1.2055 |
|  | rlcv-miR-rL1-11 | 1.14 |
|  | **cel-miR-243** | 1.0905; 1.0415 |
|  | mmu-miR-679 | -1.0035 |
|  | dme-miR-9b, dps-miR-9b | -1.0105 |
|  | hsa-miR-20b, mmu-miR-20b, rno-miR-20b, gga-miR-20bxla-miR-20, xtr-miR-20b | -1.119 |
|  | hcmv-miR-US25-2-5p | -1.1675 |
|  | osa-miR441a-, osa-miR441b-, osa-miR441c | -1.4745 |
|  | mmu-miR-291a-5p-291b-5p, rno-miR-291-5p | -1.526 |

**§**All miRNA names generated from the miRNA expression array are listed, regardless of whether the miRNA sequences are identical amongst species.

*All fold-changes (FC) are listed as the log_2_ value in order from most upregulated to most downregulated for a single biological replicate, within the restrictions of FDR <0.05 and |log_2_ fold-change| >1 for each independent biological replicate. If more than one change was identified for a single miRNA, they are listed individually.

**Bold** miRNA names indicate those miRNAs found in more than one biological replicate.
