# Supplementary material for: miR-206 integrates multiple components of differentiation pathways to control the transition from growth to differentiation in rhabdomyosarcoma cells
Source: Skelet Muscle. 2012 Apr 29;2:7. doi: 10.1186/2044-5040-2-7 (PMC3417070; doi:10.1186/2044-5040-2-7)
Supplement: Additional file 8 — Table S3.Top GO categories of genes regulated by RUNX1, ZNF238, and miR-206. [file 2044-5040-2-7-S8.doc]

**Supplemental Table S3.** Top GO Categories of genes ­­­­upregulated by RUNX1, ZNF238 and miR-206.

|  | **GO Term^a^** | **GO Category ID^b^** | **P-value^c^** | **GO Category Size^d^** | **Gene Count^e^** |
| --- | --- | --- | --- | --- | --- |
| *RUNX1-regulated genes* |  |  |  |  |  |
|  | **Muscle filament sliding** | GO:0030049 | 1.49E-19 | 30 | 18 |
|  | **Actin-mediated cell contraction** | GO:0070252 | 1.49E-19 | 30 | 18 |
|  | **Actin filament-based movement** | GO:0030048 | 5.53E-18 | 40 | 19 |
|  | Heart process | GO:0003015 | 5.77E-12 | 51 | 16 |
|  | **Muscle organ development** | GO:0007517 | 1.05E-09 | 175 | 25 |
|  | Blood circulation | GO:0008015 | 1.33E-09 | 138 | 22 |
|  | System process | GO:0003008 | 1.57E-08 | 537 | 43 |
|  | Striated muscle cell differentiation | GO:0051146 | 4.53E-08 | 78 | 15 |
|  | Sarcomere organization | GO:0045214 | 1.17E-07 | 9 | 6 |
|  | Cardiac muscle contraction | GO:0060048 | 1.50E-07 | 20 | 8 |
| *ZNF238-regulated genes* |  |  |  |  |  |
|  | **Muscle filament sliding** | GO:0030049 | 1.45E-10 | 30 | 11 |
|  | **Actin-mediated cell contraction** | GO:0070252 | 1.45E-10 | 30 | 11 |
|  | **Actin filament-based movement** | GO:0030048 | 4.84E-09 | 40 | 11 |
|  | **Muscle organ development** | GO:0007517 | 4.13E-08 | 175 | 20 |
|  | Cell differentiation | GO:0030154 | 9.43E-08 | 1241 | 64 |
|  | Developmental process | GO:0032502 | 1.22E-07 | 2251 | 97 |
|  | System process | GO:0003008 | 1.91E-07 | 699 | 43 |
|  | Muscle contraction | GO:0006936 | 6.90E-07 | 109 | 14 |
|  | Muscle cell development | GO:0055001 | 7.48E-07 | 63 | 11 |
|  | Myofibril assembly | GO:0030239 | 3.40E-06 | 17 | 6 |
| *miR-206-regulated genes* |  |  |  |  |  |
|  | **Muscle filament sliding** | GO:0030049 | 1.06E-16 | 30 | 13 |
|  | **Actin-mediated cell contraction** | GO:0070252 | 1.06E-16 | 30 | 13 |
|  | **Actin filament-based movement** | GO:0030048 | 9.40E-15 | 40 | 13 |
|  | Muscle cell development | GO:0055001 | 6.10E-12 | 63 | 13 |
|  | Heart process | GO:0003015 | 7.75E-12 | 51 | 12 |
|  | Striated muscle cell differentiation | GO:0051146 | 1.06E-10 | 78 | 13 |
|  | Cardiac muscle contraction | GO:0060048 | 2.27E-10 | 20 | 8 |
|  | Sarcomere organization | GO:0045214 | 8.30E-10 | 9 | 6 |
|  | Skeletal muscle contraction | GO:0003009 | 1.61E-08 | 13 | 6 |
|  | **Muscle organ development** | GO:0007517 | 3.98E-08 | 152 | 14 |

**Bold** categories indicate those that are common between RUNX1, ZNF238, and miR-206.

^a^The name of the indicated GO category.

^b^The ID number associated with the named GO category (BP ontology).

^c^P-value associated with the GO category.

^d^The total number of genes associated with the GO category.

^e^The number of genes contained within the category that were found to be significantly regulated in the analysis.
