# Supplementary material for: miR-206 integrates multiple components of differentiation pathways to control the transition from growth to differentiation in rhabdomyosarcoma cells
Source: Skelet Muscle. 2012 Apr 29;2:7. doi: 10.1186/2044-5040-2-7 (PMC3417070; doi:10.1186/2044-5040-2-7)
Supplement: Additional file 9 — Table S4.Select potential regulators of myogenesis affected by RUNX1, ZNF238, and miR-206. [file 2044-5040-2-7-S9.doc]

**Supplemental Table S4.** Select potential regulators of myogenesis affected by RUNX1, ZNF238, and miR-206.

|  | **Gene Symbol** | **RUNX1 FC (log_2_)^a^** | **ZNF238 FC (log_2_)** | **miR-206 FC (log_2_)** |
| --- | --- | --- | --- | --- |
| *Strong RUNX1 regulation* |  |  |  |  |
|  | MYOG | **2.01** | 1.28 | 1.17 |
|  | MEF2C | **1.81** | 1.10 | 0.84 |
|  | MEF2D | **1.50** | 0.37 | 0.69 |
| *Strong ZNF238 regulation* |  |  |  |  |
|  | MYCN | -0.71 | **-3.34** | -1.29 |
|  | RCOR2 | -1.11 | **-2.03** | -0.86 |
|  | HEYL | -0.69 | **-1.95** | -0.24 |
|  | HES6 | -0.23 | **-1.63** | -0.39 |
|  | E2F2 | -0.43 | **-1.67** | -0.01 |
|  | HEY1 | -0.53 | **-1.05** | 0.23 |
|  | HES1* | -1.09 | **-0.72** | -0.41 |
| *Strong miR-206 regulation* |  |  |  |  |
|  | NOTCH3 | -0.39 | -1.5 | **-1.75** |
|  | DLL3 | -2.07 | -1.88 | **-1.93** |

*Though it didn't reach the 2-fold change cut-off, *HES1* was included in this table for its known role in RMS.

^a^All fold-changes (FC) are reported as the log_2_ value.

**Bold** numbers indicate the fold-change in the analysis that originally identified the genes as being of potential interest.
